# Defining the Plasticity of Transcription Factor Binding Sites by Deconstructing DNA Consensus Sequences: The PhoP-Binding Sites among Gamma/Enterobacteria

**DOI:** 10.1371/journal.pcbi.1000862

**Published:** 2010-07-22

**Authors:** Oscar Harari, Sun-Yang Park, Henry Huang, Eduardo A. Groisman, Igor Zwir

**Affiliations:** 1 Department of Computer Science and Artificial Intelligence, University of Granada, Granada, Spain; 2 Department of Psychiatry, Washington University School of Medicine, St. Louis, Missouri, United States of America; 3 Department of Molecular Microbiology, Washington University School of Medicine, St. Louis, Missouri, United States of America; 4 Howard Hughes Medical Institute, Washington University School of Medicine, St. Louis, Missouri, United States of America; University of British Columbia, Canada

## Abstract

Transcriptional regulators recognize specific DNA sequences. Because these sequences are embedded in the background of genomic DNA, it is hard to identify the key *cis*-regulatory elements that determine disparate patterns of gene expression. The detection of the intra- and inter-species differences among these sequences is crucial for understanding the molecular basis of both differential gene expression and evolution. Here, we address this problem by investigating the target promoters controlled by the DNA-binding PhoP protein, which governs virulence and Mg^2+^ homeostasis in several bacterial species. PhoP is particularly interesting; it is highly conserved in different gamma/enterobacteria, regulating not only ancestral genes but also governing the expression of dozens of horizontally acquired genes that differ from species to species. Our approach consists of decomposing the DNA binding site sequences for a given regulator into families of motifs (*i.e.*, termed submotifs) using a machine learning method inspired by the “*Divide & Conquer*” strategy. By partitioning a motif into sub-patterns, computational advantages for classification were produced, resulting in the discovery of new members of a regulon, and alleviating the problem of distinguishing functional sites in chromatin immunoprecipitation and DNA microarray genome-wide analysis. Moreover, we found that certain partitions were useful in revealing biological properties of binding site sequences, including modular gains and losses of PhoP binding sites through evolutionary turnover events, as well as conservation in distant species. The high conservation of PhoP submotifs within gamma/enterobacteria, as well as the regulatory protein that recognizes them, suggests that the major cause of divergence between related species is not due to the binding sites, as was previously suggested for other regulators. Instead, the divergence may be attributed to the fast evolution of orthologous target genes and/or the promoter architectures resulting from the interaction of those binding sites with the RNA polymerase.

## Author Summary

The diversity of life forms frequently results from small changes in the regulatory systems that control gene expression. These changes often occur in *cis*-elements relevant to transcriptional regulation that are difficult to discern, as they are short, and are embedded in a genomic background that does not play a direct role in gene expression, or that consists of disparate sequences such as those from horizontally acquired genes. We devised a machine-learning method that significantly improves the identification of these elements, uncovering families of binding site motifs (*i.e.*, “submotifs”), instead of a single consensus recognized by a transcriptional regulator. The method can also incorporate other *cis*-elements to fully describe promoter architectures. Far from being just a computational convenience, ChIP-chip and custom expression microarray experiments for the PhoP regulon validated the high conservation and modular evolution of submotifs throughout the gamma/enterobacteria. This suggests that the major cause of divergence between species is not due to the binding sites, as was previously suggested for other regulators. Instead, the divergence may be attributed to the fast evolution of orthologous and horizontally-acquired target genes, and/or to the uncovered promoter architectures governing the interaction between the regulator and the RNA polymerase.

## Introduction

Whole genome sequences, as well as microarray and chromatin inmunoprecipitation with array hybridization (ChIP-chip) data provide the raw material for the characterization and understanding of the underlying regulatory systems. It is still challenging, however, to discern the sequence elements relevant to differential gene expression, such as those corresponding to the binding sites (BSs) of transcriptional factors (TFs) and RNA polymerase (RNAP), when they are embedded in the background of genomic DNA sequences that do not play a role in gene expression [1]. This raises the question: how does a single regulator distinguish promoter sequences when affinity is a major determinant of differential expression? Also, how does a regulator evolve given that there appears to be a non-monotonic co-evolution of regulators and targets [2]–[4]?

Methods that look for matching to a consensus pattern have been successfully used to identify BSs in promoters controlled by particular TFs [5]–[7]. Tools for motif discovery are designed to find unknown, relatively short sequence patterns located primarily in the promoter regions of genomes [1]. Because these searches are performed in a context of short signals embedded in high statistical noise, current tools tend to discard a relevant number of samples that only weakly resemble a consensus [8]. Moreover, the strict cutoffs used by these methods, while increasing specificity, display lower sensitivity [6], [9] to weak but still functional BSs. Because the consensus motif reflects a single pattern derived by averaging DNA sequences, it often conceals sub-patterns that might define distinct regulatory mechanisms [10]. Overall, the use of consensuses tends to homogenize sequence motifs among promoters and even across species [11], [12], which hampers the discovery of key features that distinguish co-regulated promoters within and across species.

**Figure 1:**
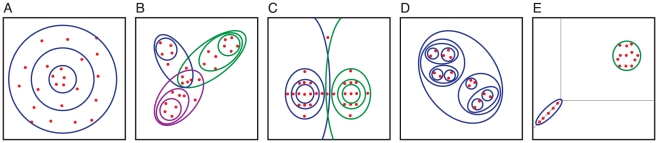
Characterization of clustering methods. Generic data (red dots), clustering partitions (circle and ovals), and their membership scopes as defined by their most characteristic distance metrics and algorithm are represented. **A**) Substractive clustering [30] applied to sparsely distributed datasets. **B**) Crisp clustering [28] (*e.g.*, K-means) applied to fuzzy datasets. **C**) Probabilistic [28] (*e.g.*, Expectation-Maximization) or fuzzy clustering (*e.g.*, C-means) [31] applied to datasets with several outliers. **D**) Hierarchical clustering [33] applied to datasets displaying many patterns with small extent. **E**) Feature selection clustering [14], [34] applied to datasets harboring patterns involving different sets of features.

To circumvent the limitations of consensus methods [1], we decomposed BS motifs into sub-patterns [13], [14] by applying the classical *Divide & Conquer* (*D&C*) strategy [15], [16]. We then compared different forms of decomposed BS motifs of a TF into families of motifs (*i.e.*, “submotifs”) from a computational clustering perspective (Figure 1). In so doing, we extracted the maximal amount of useful genomic information through the effective handling of the biological and experimental variability inherent in the data, and then combined them into an accurate multi-classifier predictor [13], [17]. Although there is a computational usefulness of the submotifs [13], [14], it was not clear if these families of motifs were just a computational artifact or if they could provide insights into the regulatory process carried out by a regulator and its targets.

To address this problem, we evaluated the ability of the submotifs to characterize gene expression both within and across genomes. First, we used submotifs to distinguish between functional and non-functional BSs in genome-wide searches using a combination of ChIP-chip and custom expression microarray experiments (Nimblegen tiling arrays). Then, we determined the evolutionary significance of the submotifs by calculating their rate of evolution [18], [19] and mapping the gain and loss events along the phylogenetic tree of gamma/enterobacteria. The interspecies variation of orthologous genes, the conservation of the regulatory protein, as well as the *cis*-features conforming the promoter architecture allowed us to evaluate the major causes of divergences between species [4], [20].

We applied our approach to analyze the genes regulated by the PhoP/PhoQ two-component system, which mediates the adaptation to low Mg^2+^ environments and/or virulence in several bacteria species including *Escherichia coli* species, *Salmonella* species, *Shigella* species, *Erwinia* species, *Photorhabdus* and *Yersinia* species (See [21] for a review). Two-component systems represent the primary signal transduction paradigm in prokaryotic organisms. Although proteins encoded by these systems are often well conserved throughout different bacterial species [2], [3]), regulators like PhoP differentially control the expression of many horizontally-acquired genes, which constitute one of the major sources of genomic variation [22].

## Results

### A single cluster of BS sequences for a TF cannot fully describe the entire repertoire of BS recognition

A diverse collection of useful tools [1] have been developed to analyze DNA sequences bound by a TF and to discover recurrent patterns of nucleotides, termed motifs, that differ from the genome's background. These tools vary in their search algorithms and measurements used to characterize a candidate motif [6], [23], [24], which ultimately consists of a single cluster of sequences [25]. Evaluation of three of these tools (*i.e.*, Consensus [9], MEME [24] and AlignACE [23]) demonstrated that a single cluster is not sufficient to appropriately describe the direct-repeat BSs of the PhoP protein (Table S1), which as a global regulator either directly or indirectly controls ∼5% of the genes in the Gram-negative pathogen *Salmonella enterica* serovar Typhimurium LT2 [26], including products with different functions such as transcriptional regulation, Mg^2+^ transport, and modification of membrane components [26]. Moreover, these methods were not able to describe the inverted-repeat BSs of the well known cyclic AMP receptor protein (CRP) regulon in *Escherichia coli*, which is characterized by ca. 150 instances collected in the RegulonDB database [27] (Table S2). This is because searches for overrepresented sequences are performed in a context of short signals embedded in high statistical noise. A single cluster tends to discard the samples that only weakly resemble its centroid, which is represented by a consensus pattern [8].

### The decomposition of a TFBS into a family of motifs overcomes limitations of a single cluster to describe a regulon

Traditional clustering approaches (See [28] for a review), involving multiple clusters, can be applied to find commonalities among TFBS sequences, thus, circumventing the limitations of a single motif cluster [25], [29]. There are, however, several limitations of the clustering methods that might be exacerbated by attempting to group short and noisy DNA sequences together (Figure 1). Those limitations may include retrieving redundant sub-patterns when applying substractive clustering [30] to sparsely distributed data (Figure 1A), because sequentially retrieved clusters, having similar centroids, may reflect the same pattern supported by a decreasing number of instances; sub-patterns that contain unrelated data when applying crisp clustering [31] to datasets displaying fuzzy patterns (Figure 1B), because instances are forced to belong to one and only one cluster even if they substantially differ from the cluster centroid or partially match with more than one cluster; vague or incorrect number of sub-patterns when applying probabilistic or fuzzy clustering [28] to data containing outliers (Figure 1C), because the sum of membership of an instance to one or more clusters must equal to one even if it partially belongs to one cluster (*i.e.*, membership <1) and does not belong to any other complementary cluster (*i.e.*, possibilistic clustering [28], [32]); many sub-patterns with small extent [33], as it is easier to explain smaller data subsets than those that constitute a significant portion of the dataset, when using a non-hierarchical clustering organization (Figure 1D); or finally, global sub-patterns identified by the full set of features [14], [34] when different features might be relevant for distinct clusters (Figure 1E).

We applied these clustering methods to characterize the BSs of a TF and established that different clustering methods recover distinct position-dependent sub-patterns. For example, the well characterized TFBSs of the CRP regulator [27], [35] exhibit different variants of its canonical BS motif (Text S1 and Figure S1). Despite their differences, the clustering methods improved the classification of the PhoP (Table S1) and CRP regulons (Text S2 and Tables S2, S3), even when they were integrated into a simple classifier (See Materials and Methods).

### A *Divide & Conquer* approach designed to identify biologically meaningful BSs

**Figure 2:**
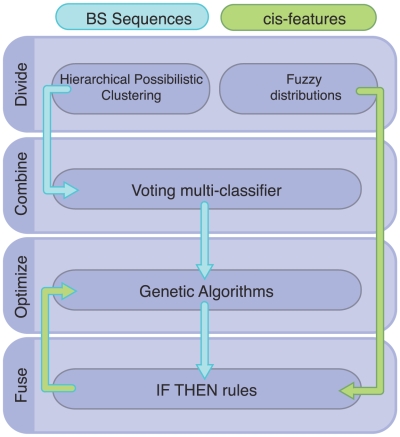
A Divide & Conquer method to clarify transcription factor binding sites. *D&C* consists of 4 phases. 1) Divide BS sequences into submotifs by using the possibilistic implementation of the fuzzy clustering method, and then organizing them hierarchically. The submotifs are encoded into PWMs (*i.e.*, Consensus, Meme and AlignAce) and the distances from a TFBS to the RNAP into distributions represented as fuzzy sets. 2) Combine PWMs from submotifs into a multi-classifier. 3) Optimize the accuracy and complexity of the multi-classifier by using genetic algorithms. 4) Fuse different *cis*-features into fuzzy IF THEN rules, where the antecedents are the conjunction of the individual features, and the consequents are the prediction of a TFBS.

We used a machine learning method, inspired by the classical *D&C* approach [15], that integrates the advantages and overcomes some of the limitation of the methods described above (Figure 2). First, we grouped DNA sequences using a possibilistic fuzzy clustering method [28], [32]. The fuzzy-based algorithm allows a DNA sequence to belong to, or be aligned with, more than one cluster of sequences with distinct degrees of membership (*i.e.*, 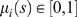, where *s* is a DNA sequence and *i* is a cluster), but uses the probabilistic constraint that states that the sum of membership degrees of each data sequence equals to one (*i.e.*, 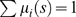). Because outliers, as well as membership degrees generated by the fuzzy-based algorithms sometimes deviate the original dissimilarities among observations, we applied a fuzzy clustering variant termed “possibilistic” that permits a sequence to partially belong to one cluster (*i.e.*, 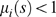)), and not to the others (*i.e.*, 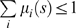). Second, we hierarchically organized them as families [36]. Third, we encoded these groups into submotifs using position weight matrix (PWM) methods [1]. Fourth, we combined them into a voting multi-classifier [17], which characterizes a DNA sequence as a TFBS by utilizing the combined strength in terms of specificity and sensitivity of the submotifs.

The individual and cooperative contribution of each submotifs to the multi-classifier are optimized using Genetic Algorithms (GA) [37]. This multi-objective [38] optimization involves the identification of thresholds that increases the sensitivity to weak sites without losing specificity, as well as minimizing the number of submotifs, thus reducing the complexity of the final classifier. In addition to combining submotifs, the classifier is versatile enough to incorporate other *cis*-promoter features constraints (*e.g.*, genome location and orientation with resperc to the RNAP BS [39]–[41]).

### Scrutinizing the PhoP regulon

**Figure 3:**
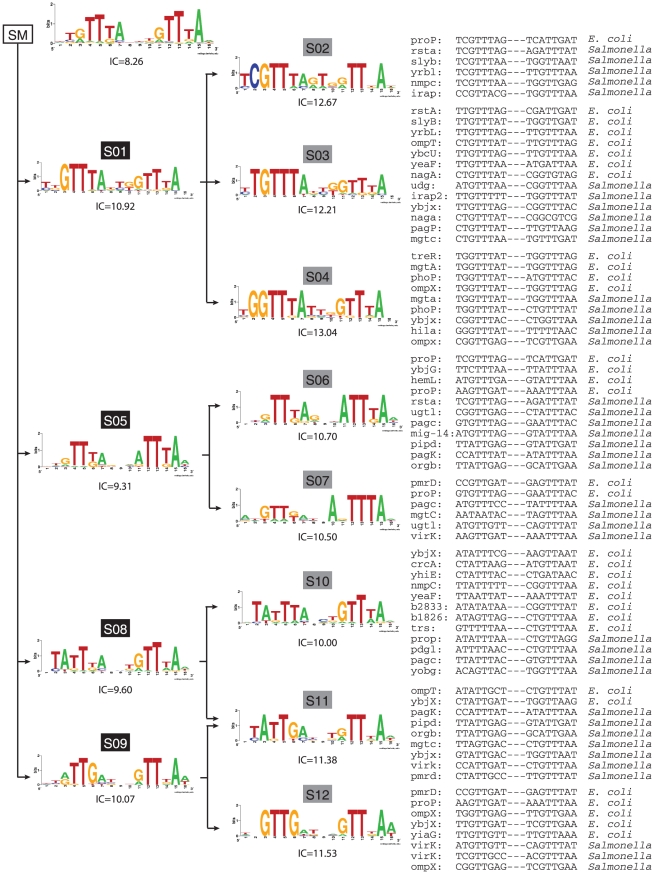
Families of PhoP BSs submotifs in *E. coli* K-12 and *S. typhimurium*. The tree represents the hierarchical organization of PhoP submotifs; which are represented by their logos (three nucleotides between the direct repeat tandems are omitted). The root corresponds to the consensus (single) motif (left panel), while general and specific submotifs are ordered from left to right. Sequences conforming to each specific submotifs (gray boxes) and their genomic source are listed on the right panel. The information content of each submotif is displayed below the logos (*i.e.*, the higher the more informative).

We studied the PhoP BSs found in *E. coli* K-12 and *S. typhimurium* that have been reported in the literature [26], [42], as well as our previous work [41]. As a result, we collected 69 DNA sequences corresponding to PhoP BSs, where 31 are BSs from 25 *E. coli* genes and 38 are BSs from 28 *Salmonella* genes. Some promoters have more than one BS, and 14 genes are orthologous among these two species [43]. BSs corresponding to promoters for orthologous genes are considered as independent examples, where every sequence instance is considered equally important. For example, the sequences corresponding to the PhoP BSs in the promoters of the *E. coli* and *Salmonella phoP* orthologous genes are similar to each other [42], [44], and both sequences belong to the same submotif (Figure 3). In contrast, the PhoP BS sequences in the promoters of the *E. coli* and *Salmonella slyB* genes are grouped into different submotifs (Figure 3), despite the orthology of the genes [44]. Furthermore, PhoP binds to the promoter of the *Salmonella ugd* gene, but it does not bind to the corresponding promoter in the *E. coli ugd* gene, despite these genes being 88% identical [45], [46].

We applied a hierarchical possibilistic clustering as described above, and identified 12 PhoP submotifs organized into families (Figure 3). For example, the sequences assigned to submotif S01, were also assigned to more specific submotifs S02, S03, and S04, which correspond to different sub-patterns (Figure 3). The logo representation illustrates the differences between sub-patterns. For example, submotif S05, present in the *proP*, *ybjX* and *mig*-14 promoters, has a strong pattern for the second tandem that differs from the canonical S01 submotif, present in the *mgtA* and *phoP* promoters, which harbors a strong pattern in both direct repeats. Because alignments of short DNA sequences are ambiguous [6], we allow a BS sequence to be aligned with, or belong to more than one submotif (*i.e.*, fuzzy clustering). Therefore, redundant (similar) sequences do not bias the alignment toward their own features, because there are multiple submotifs instead of an unique-alignment/motif [47].

### Submotifs improve the classification performance of PhoP BSs

We encoded the 12 submotifs into a computational predictor termed “multi-classifier” [17], where the classification of a sequence as a TFBS is derived from its similarity to one or more submotifs. To calculate the performance of the multi-classifier we considered 772 BS of other TFs as negatives examples in promoter sequences reported in the RegulonDB database [27]. The multi-classifier was optimized for best global Correlation Coefficient (CC), or its modified version Standardized Correlation Coefficient (SCC) for unbalanced numbers of positive and negative examples [1], by adjusting the individual thresholds of the PWMs corresponding to the submotifs. Encoding the submotifs by Consensus improved SCC by 29% (*i.e.*, 0.835 vs. 0.547, *F*-statistic *p-value*<3.91e-05), and CC by 23% (*i.e.*, 0.885 vs. 0.653, *F*-statistic *p-value*<1.84e-06) compared to those obtained by the single motif model. This enhancement is due to the recovery of 25 BSs that were not detected by the single motif approach (*i.e.*, 57 vs. 32 BSs) while predicting only one additional false positive.

Separately, we tested our results utilizing the same number of negative examples, but derived from random sequences generated according to a Markov model based on intergenic sequences of *E. coli* and *Salmonella* genomes [48]. The SCC and CC obtained by the multi-classifier in a random background model were improved by an additional 7% (*i.e.*, 36%) and 4% (*i.e.*, 27%), respectively. (In the following, we use BSs of other TFs as negative examples because it is a more stringent criterion than using random sequences). In addition, the multi-classifier was able to capture more than one BS per gene (Figure 3), which are located upstream of, overlapping with, or downstream of the RNA polymerase binding site [27], [42]. This suggests a possible combination of activation and repression PhoP boxes within the same PhoP-regulated promoters [49].

We obtained similar results encoding submotifs by MEME, improving SCC by 50% (*i.e.*, 0.924 vs. 0.613, *F*-statistic *p-value*<3.40e-04) and CC by 31% (*i.e.*, 0.928 vs. 0.707, *F*-statistic *p-value*<6.47e-04). Similarly, by using AlignACE, we observed an improvement of 25% for the SCC (*i.e.*, 0.883 vs. 0.708, *F*-statistic *p-value*<3.81e-04) and 21% for the CC (*i.e.*, 0.870 vs. 0.718, *F*-statistic *p-value*<0.006). (See Text S3 and Table S4 for cross-validation analysis). These results include the evaluation of different clustering methods (“Divide phase”) (Figure S2), which suggest that all clustering methods examined improved the classification of BSs, even with a simple integration into a classifier (“Conquer phase”). However, the hierarchical possibilistic clustering demonstrates the best encoding (*i.e.*, minimizes the differences among the information content of the submotifs) and the most interpretable model (Figure 3 and Figure S3). (See Text S2, and Tables S2, S3 for similar results for the CRP regulon).

### The submotifs identified are necessary to describe the PhoP BSs fully

**Figure 4:**
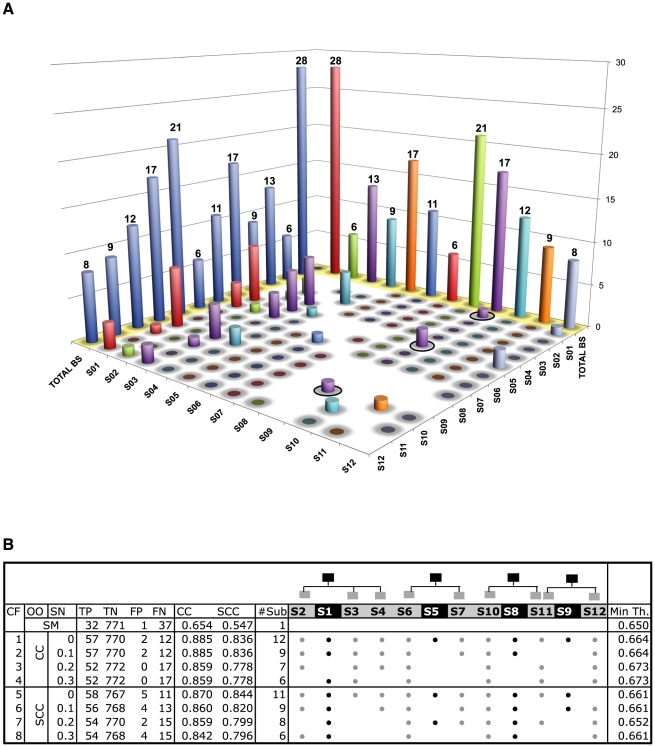
Sensitivity analysis of *D&C* parameters. **A**) Leave-one-submotif-out cross-validation of the PhoP BSs. Each color bar represents a different submotif, and its height shows the number of BSs that it recognizes. Shaded in yellow at the back edge, the height of each bar indicates the number of BSs originally clustered in the corresponding submotif. The inner bars for a given submotif indicate the number of its BSs that are also recognized by other submotifs (*e.g.*, S09 was originally composed by 17 BSs, 1 of these BSs is also recovered S01 (black circle); 2 BSs are also recovered by S05; and 1 BS is also recovered by S10). The diagonal was omitted in its corresponding place for clarity purposes, but drawn in the back as blue bars. The number of BSs recognized by multiple submotifs (i.e., intersections among submotifs) is low as shown by the low height of the inner bars (Table S5). **B**) Optimal configurations of submotifs encoded by Consensus PWMs obtained from GA optimization of the number and thresholds of submotifs (CF). The fitness function was calculated by either SCC or CC measurements (OO). Different selection pressures (SN) where used as initial constrains (See 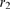 parameter in Materials and Methods). TP/TN and FP/FN stand for true/negative and positive/negative predicted values, respectively. #Sub indicates the number of submotifs effectively employed, columns S1 to S12 represent the submotifs organized as families. Dots at the columns (black: general submotif; white: specific submotif) indicate that the corresponding submotif was selected by the optimization process for that configuration (rows). Min Th. corresponds to the minimum learned threshold. SM shows the results obtained by the single motif (See Figure S5 for similar results using MEME and AlignACE methods).

We evaluated whether the families of submotifs identified in this work were necessary to describe the PhoP regulon, and determined that both general (S01, S05, S08 and S09) and specific (S02–S04, S06–S07, S10–12) submotifs contributed to the classification of the PhoP BSs. For example, the S01 is a generalization of its dependent submotifs (Figure 3), but its PWM does not recover BSs for the *Salmonella iraP*, *nagA* and *ybjX* promoters, which are detected by the more specific PWMs of the S02, S03 and S04 submotifs, respectively. Similarly, the PWM of the S05 submotif recognizes neither the PhoP BSs in the *Salmonella mgtC* and *virK* promoters nor the BSs in the *ybjX* promoter of *E. coli*, which are only recovered by the more specific PWMs of the S06 and S07 submotifs (Figure 3). An iterative leave-one-submotif-out analysis [50] showed that the families of submotifs identified in this work are necessary to describe the PhoP regulon (Figure 4A and Table S5). For example, the BSs recognized by the PWMs of the S08 family of motifs are not retrieved by any other family of motifs (Figure 4A). Remarkably, and despite the non-significant overlap among submotifs (Hypergeometric test [51], *p-value*>0.05), few BSs originally grouped together by one family of submotifs were recognized by another family, as witnessed in fuzzy clustering (Figure 4A).

The sensitivity and specificity of the multi-classifier depends not only on the thresholds of the individual submotifs, but also on its complexity, which is determined by the number of submotifs used. Because the more complex the classifier the greater the chances of overfitting the data, we incorporated another constraint into the optimization process to minimize the complexity of the multi-classifier (*i.e.*, multi-objective optimization [38]). This resulted in several optimal configurations of the multi-classifier (See Text S3 and Figure S4 for a detailed analysis of the optimization process). Notably, all optimal configurations for all PWM methods preserve at least one member of each family of submotifs (Figure 4B and Figure S5).

### Submotifs distinguish functional PhoP BSs in genome-wide analysis

We investigated the ability of the proposed multi-classifier that encodes families of submotifs to detect PhoP BSs in whole genome sequences by screening the intergenic and coding regions of the *S. typhimurium* strain LT2 genome. We used the data described above to perform a genome wide prediction of the PhoP regulation in *Salmonella*, including its binding in promoter and coding regions, as well as the corresponding gene transcription. Our analysis considered each gene harboring an >20 bp intergenic region, as well as the head of its corresponding operon (generously predicted in *Salmonella* and provided by H. Salgado, RegulonDB [52]), as possible binding targets of the PhoP protein. In the same fashion, we used *Yersinia pestis* KIM as a test organism.

To evaluate our predictions, gene expression was measured by microarray assays of wild-type and *phoP* mutated strains, while promoter occupancy of the PhoP protein was measured by a ChIP-chip assay. Based on these experimental results, we subdivided the genes into three subsets: expressed genes harboring a significant peak (*i.e.*, Log_2_-ratio of the ChIP-chip signal intensity detected using a 500 bp sliding window) in the intergenic region corresponding to promoter binding by the PhoP protein; expressed genes without such a peak; and genes harboring a peak without exhibiting significant expression (Table S6).

**Figure 5:**
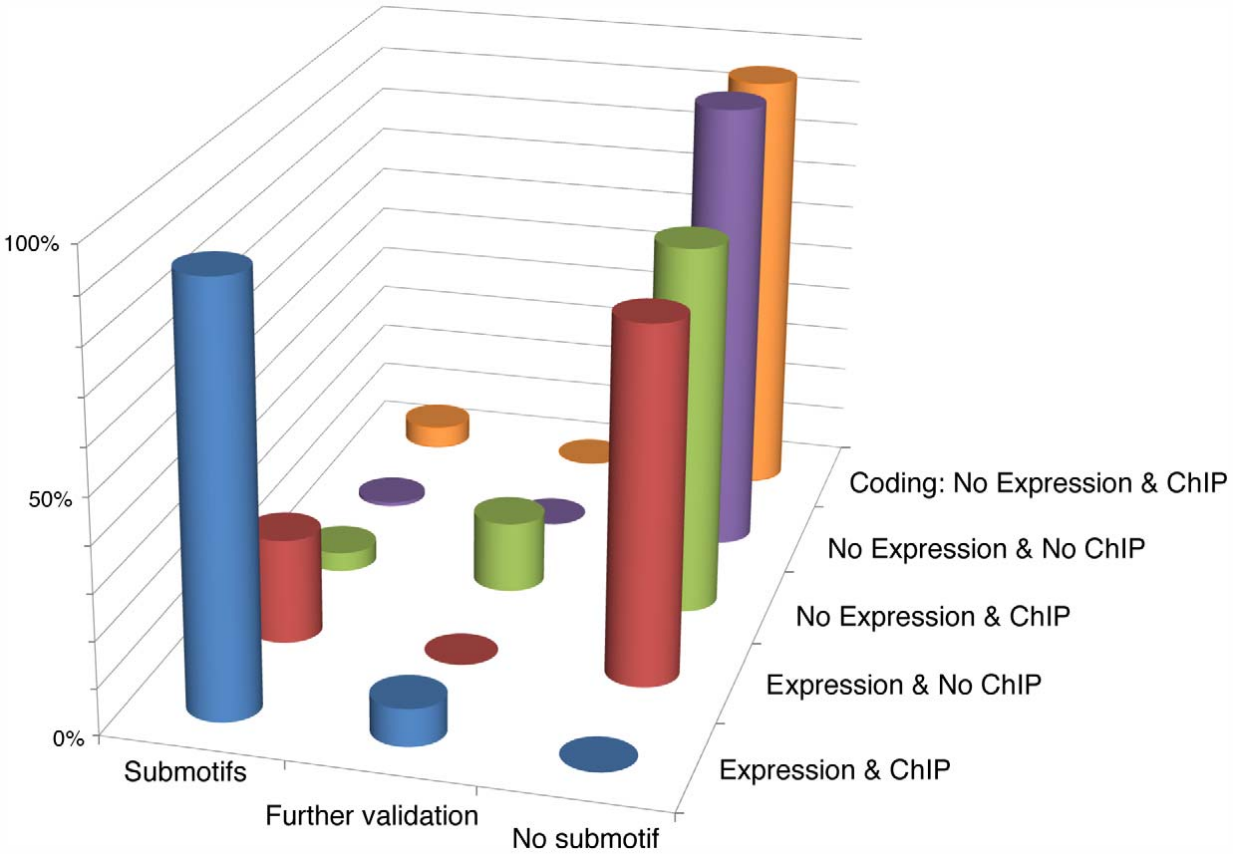
Genome-wide analysis of the *S. typhimurium* sequences using PhoP submotifs. Four categories (Y axis) of genes were identified based on the presence or absence of PhoP promoted expression as measured by the tiling array, and the presence or absence of PhoP binding as measured by ChIP experiments. The fifth category corresponds to the presence of ChIP peaks in coding regions corresponding to non-expressed genes. The height of the bars represents the percentage of presence or absence of PhoP BSs identified by the submotifs (X axis), as well as by the need of further validation of the results (See Text S4 for details), within the intergenic regions of genes belonging to the first four categories, and the coding regions corresponding to the fifth category. The analysis performed on the first four categories considered each gene harboring an intergenic region with >20 bp, and/or the head of its corresponding operon as possible binding targets of the PhoP protein. (See Table S7 for gene/operon numeric data).

We detected PhoP BSs in 34 genes displaying significant expression and ChIP scores (Figure 5 and Table S7). We did not detect BSs in 54 of the total 70 expressed genes without significant ChIP peaks. Of these 54 most are members of operons whose first gene harbors a PhoP BS or are genes known to be indirectly regulated by PhoP [53], [54] (See Text S4). However, we did find BSs in the remaining 16 genes (Figure 5). Although most of thees 16 genes have been proposed to be indirectly regulated by PhoP via another regulatory protein(s) [55], we demonstrated that PhoP directly bound to these promoters [41], even though ChIP gives negative results. Overall, we identify 50 genes whose promoters harbor PhoP binding sites with detectable effects upon transcription.

We did not detect PhoP BSs in the intergenic regions adjacent to 57 genes (Figure 5 and Table S7) that harbor ChIP peaks but give no evidence of PhoP-dependent transcription. At this point we do not know if PhoP binds at these sites. Moreover, we did not find PhoP BSs in 95% of the 266 significant ChIP peaks located in coding regions of 121 genes (Peaks with even slightly different interval positions from three replicas of the ChIP-chip assay were considered to be different). Further experiments will be required to determine if the 5% of the genes that did contain sequences resembling submotifs with corresponding ChIP peaks are indeed bound by PhoP [8] (See Text S4 for a detailed analysis) and the function of the PhoP binding, if any [8], [35]. In addition, our multi-classifier did not find PhoP BSs in the 99% of the >20 bp intergenic regions corresponding to 3327 genes without PhoP-promoted transcription and without ChIP peaks, thus the False Discovery Rate (FDR) of the method is 1% [56] (Figure 5 and Table S7).

At least 14 promoters appear to harbor more than one PhoP BS. These BSs usually correspond to the S05, S08 and S09 families of motifs. Most of these promoters drive the transcription of horizontally-acquired genes in *Salmonella* (Figure 3). We found that at least 27 of 34 of these BSs are functionally associated with PhoP–activated genes (data not shown), and they are rarely detected by the single motif approach (*i.e.*, only 5% of them). 13 of the BSs are located closer together than the minimum length of a ChIP-chip peak, and thus, they are hard to detect by that technique. In sum, the use of submotifs improves the identification of members of the PhoP regulon, and to provide a snapshot of the PhoP regulation in the whole *Salmonella* genome.

### Evolution of PhoP submotifs among gamma/enterobacteria

Based on theoretical grounds, it has been suggested that the rate of evolution at each position in a BS motif of a regulatory protein is a function of the frequencies in the corresponding PWM [18], [19]. An actual functional binding site must remain recognizable by the regulatory protein, and therefore is constrained from freely mutating and would therefore evolve slower than the surrounding sequence [18], [19], provided that the regulatory protein is not changing as well (the DNA-binding helix in the PhoP protein, like other regulators of two-component systems [2], has not changed significantly (*e-value*<1E-5, expected number of false positives in a reciprocal BLAST search [43])) (Figure S6). In other words, one would expect that functional submotifs in non-coding regions to evolve slower among the gamma/enterobacteria than “background” DNA sequences [18], [19].

**Figure 6:**
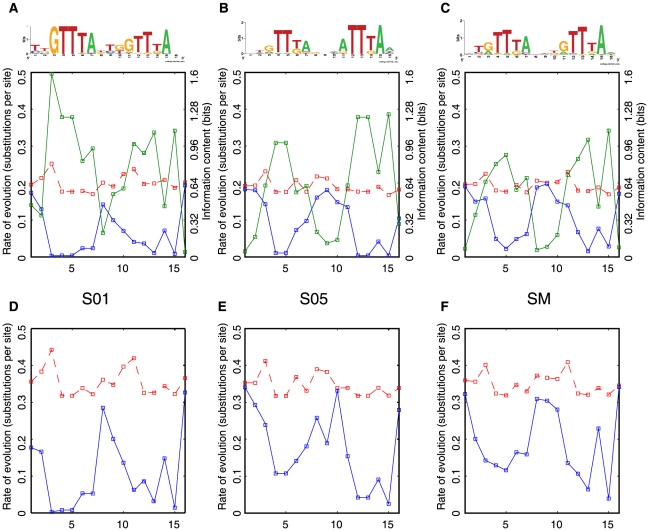
A model of evolution of the PhoP BSs based on submotifs. Rate of evolution calculated by nucleotide substitutions at each position of the PWMs corresponding to submotifs using the HB model (blue) [18], [19], compared with the background distributions using the HKY model (red) [18], [19] based on randomly selected non-coding sequences from *E. coli* K-12 and *S. typhimurium* genomes. The information content (green) is inversely correlated to the rate of evolution of submotifs. **A**) Low rates of evolution of the S01 submotif. **B**) High rates of evolution of S05 submotif. **C**) Rates of evolution of single motif. **D**) Same as in **A**), but using an AT rich non-coding intergenic regions in the background model. **E**) Same as **B**), but using an AT rich non-coding intergenic regions in the background model. **F**) Same as **C**), but using an AT rich non-coding intergenic regions in the background model.

We inferred the relative rate of nucleotide substitutions at each position in the PhoP submotifs using the model of Halpern and Bruno [19], [57] and then compared them to each other as well as against the rates of a set of aligned random non-coding regions (see Materials and Methods) (Figure 6). We found that the number of substitutions per site and the information content of the submotifs revealed a correspondence between positions of high information content and slower rates of evolution (Figure 6A–C), as expected. We also found that distinct submotifs have different rates of evolution (Figure 6A–B). For example, S01 exhibits a slower rate of evolution than S05, which is the most mutable submotif (Figure 6A–B) (*i.e.*, 0.03 vs. 0.01 Mean Square Difference (MSD), see Materials and Methods).

Given that different submotifs have different rates of evolution, there is a possibility that those submotifs with higher rates of evolution are more likely to eventually disappear [18], [19]. Interestingly, the S05 family of motifs is found primarily in promoters derived from horizontally-acquired genes, while the S01 family of submotif is mostly found in ancestral promoters. The difference between S01 and S05 families is emphasized (Figure 6A–B vs. Figure 6C–D) when these submotifs are evaluated in an AT rich background, which is the typical background of horizontally-acquired genes (Figure 6D–E) (*i.e.*, 0.09 vs. 0.03 MSD).

**Figure 7:**
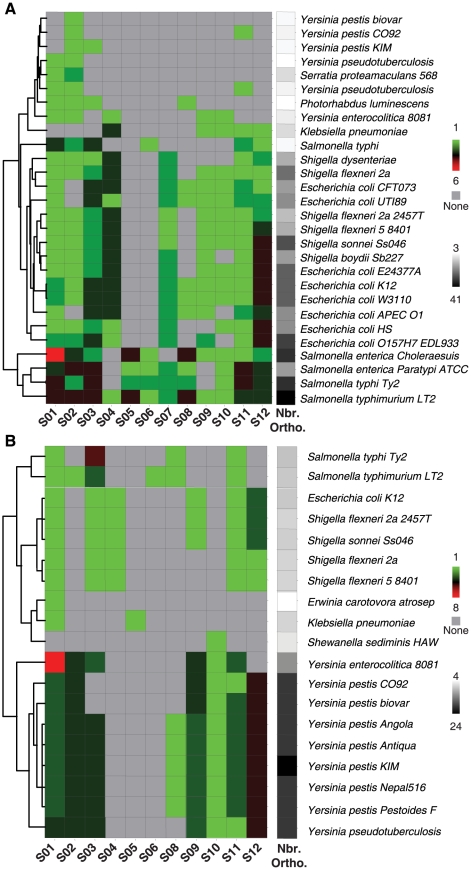
Evolution of the PhoP regulon analyzed by PhoP BSs submotifs. **A**) Distributions of submotifs identified in promoter regions of *E. coli* K-12 and *S. typhimurium* orthologous PhoP targets (left panel, green: 1, red: 6 BSs). The number of orthologous genes in each species (right panel, white: 3, black: 41 genes). **B**) Same as in **A**) but considering *Y. pestis* orthologous PhoP targets (left panel, green: 1, red: 8 BSs). The number of orthologous genes in each species (right panel, white: 4, black: 25 genes).

To test if some submotif families are better conserved than others, we examined their distribution among the gamma/enterobacteria. We first identified the presence of the submotifs in the intergenic regions of orthologous *E. coli* K-12 and *S. typhimurium* genes regulated by PhoP in the gamma/enterobacteria (*e-value*<1E-5 [43]) (Figure 7A). We found that the frequency of PhoP submotifs across the genomes is correlated (*F*-statistic; *p-value* = 2.10E-10) with the number of orthologous genes. (It should be noted that some *Yersinia* genes are xenologs rather than orthologs to those of *E. coli* or *Salmonella*, because they seem to have been acquired independently by the *E. coli*/*Salmonella* and *Yersinia* lineages [44]). This raises the possibility that more distantly related species do harbor PhoP submotif families and, consequently, genes directly regulated by PhoP that would not be detectable by a one-way search of orthologous genes.

**Figure 8:**
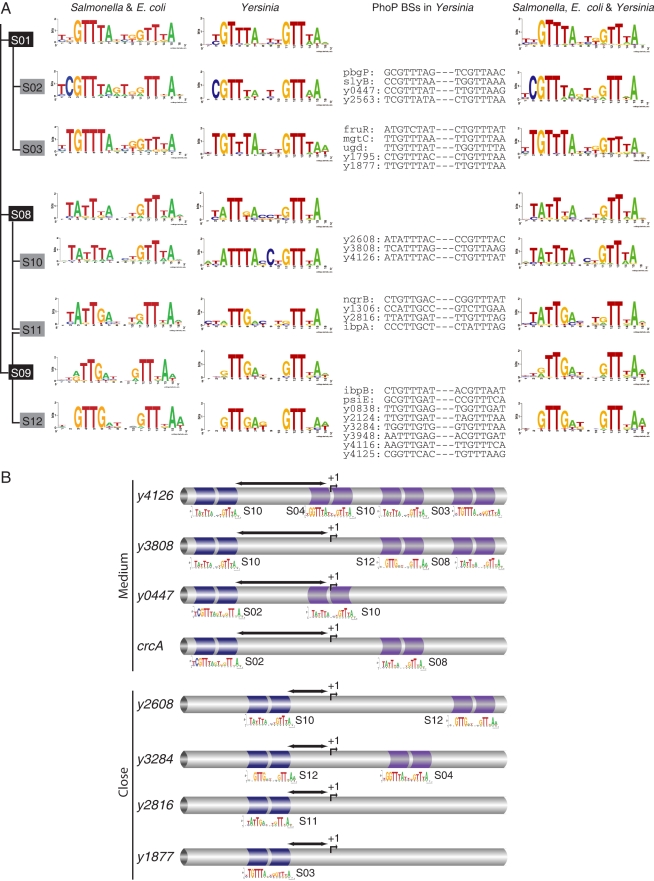
The PhoP regulon in *Yersinia pestis* KIM. **A**) PhoP BSs detected in *Y. pestis* promoters based on *E. coli* K-12 and *S. typhimurium* (lefts panel). Submotifs reconstructed from PhoP targets detected in *Yersinia* [44] (middle panel). Joined submotifs from *E. coli*, *Salmonella*, and *Yersinia* PhoP BSs (right panel). **B**) Promoter architectures and submotifs identified in PhoP-activated promoters in *Yersinia*. Schematic based on DNase I footprinting analysis [58] of the promoter regions for selected genes (blue: highest affinity; violet: lower affinity sites). BSs overlapping or downstream the TSS correspond to repression sites. *Close* and *medium* distances between PhoP BSs and the RNAP BSs are highlighted by arrows.

We therefore identified PhoP-regulated genes in *Y. pestis*, which is a more distantly related species, by using ChIP-chip and custom expression microarray experiments [58] (Table S8). Then, we searched for PhoP submotifs in the intergenic regions of the identified PhoP-regulated genes (Figure 7B). In this process we recognized the S02 and S03 submotifs in two *Salmonella* orthologous PhoP-regulated genes in *Yersinia* (Figure 7A), and also identified the S01 and the S08–S12 submotifs that would be missed by simply using a one-way search (Figure 7B and Figure 8A). However, both searches completely lacked the S05 family of submotifs (Figure 7A–B and Table S8), like the other *Yersinias*. These were genuine PhoP BSs, as was validated experimentally by DNase I footprinting analysis [58] (Figure 8B). By doing both forward (*i.e.*, orthologous *E. coli* and *Salmonella* genes regulated by PhoP) and reverse (*i.e.*, orthologous *Yersinia* genes regulated by PhoP) searches we believe we have a complete catalog of the submotifs families among these species. As in the *Salmonella* genome, there is a set of PhoP-regulated promoters in *Yersinia* harboring more than one BS (Figure 8B). These BSs were predicted by the multi-classifier based on submotifs and validated as described above [58] (Figure 8B and Table S8).

We found that the S01 family of submotifs, including the S02, S03, and S04 submotifs, is conserved in most of the analyzed species, and most of its representative submotifs are also present in the distantly-related *Yersinia* genomes (Figure 7), as was predicted by its low evolution rate (Figure 6B and Figure 6E). In contrast, the S05 family of submotifs, harboring higher rates of evolution (Figure 6A and Figure 6D), is not found in *Klebsiella*, *Erwinia, Shewanella*, *Photorhabdus*, and *Yersinia* (Figure 7). Thus, the S01 family that has a low mutation rate persists throughout the gamma/enterobacteria in contrast to the S05 family that has high mutation rate which is not present in *Yersinia* (Figure 7). In fact, if we look at species closer to *Salmonella* we see that the S05 family of motifs is also more prone to loss. The S05 family, which includes the more specific S06 and S07 submotifs, is present in most of the *Salmonella* species evaluated here, but only the S07 submotif is conserved in the closely-related *E. coli* and *Shigella* strains/species (Figure 7).

It is conceivable that instead of the mutation rate being the dominant factor, it is the rate of loss or gain of horizontally-acquired genes that determine the fate of the submotif families. If this is the case, we would expect random loss of the submotif families. This is not what we observe when analyzing families of submotifs throughout the gamma/enterobacteria (Figure 7). Yet, there are some differences in the occurrences of individual submotifs between and within close-related species (Figure 7). For example, all the submotifs are found in *E. coli* K-12, whereas the promoters of the *E. coli* UTI89, CFT073 and APEC 01 ortholog genes do not contain submotif S02. The promoters of *E. coli* O157-H7 genes do harbor this submotif but lack submotifs S08 and S10 (Figure 7). This is also true within *Salmonella*, where we see the different extent of conservation of the S04 submotif (Figure 7A), as well as the lack of the S09 submotif in *S. typhi* and *S. choleraesuis* (Figure 7B). Because submotifs with slow (*e.g.*, S02) and slightly faster (*e.g.*, S08) rates of evolution are implicated in differences between closely-related species and even within strains of the same species, it is possible that a model based on the rates of evolution could be partially obscured by the frequent and different patterns of horizontal gene acquisition among closely-related species.

### The PhoP submotif and its distance from the RNA polymerase binding site differentiates species-specific PhoP-activated promoters

TFs recognize specific sequences in promoters to activate or repress gene transcription by RNAP. The distances between these TF binding sites with respect to the RNAP BSs revealed non-random distributions in the *E. coli* genome [10], [27], [41], which indicate different classes of interactions between the two regulatory elements [4], [10]. Thus, we explored the possibility that the locations of PhoP BS in PhoP-activated promoters are different between *E. coli* K-12 and *S. typhimurium*.

**Figure 9:**
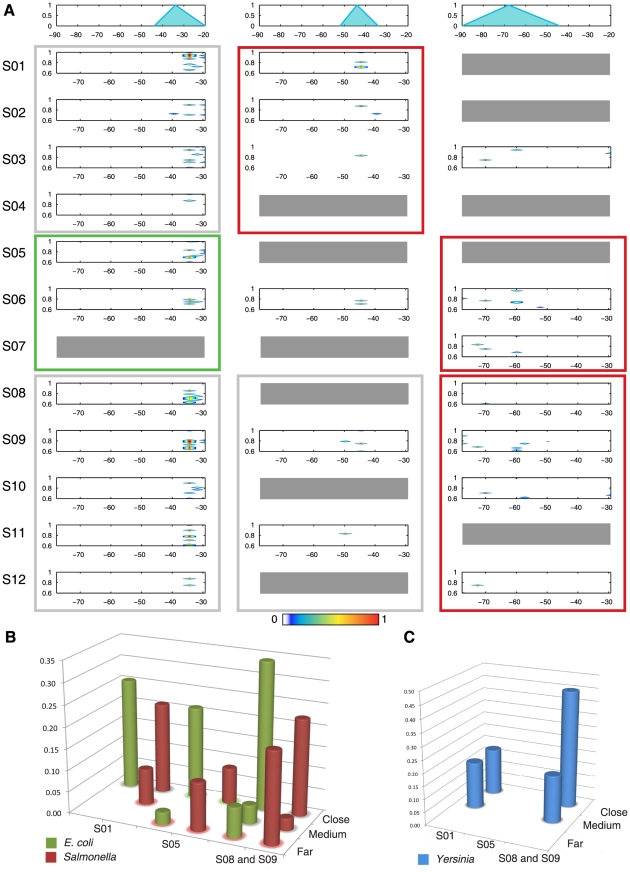
IF-THEN rules encompassing submotifs and distances between PhoP BSs and RNAP BSs. **A**) Cells correspond to IF-THEN rules. The antecedent of the rules is composed of submotifs (vertical left panel) and the distances between PhoP BSs and RNAP BSs (horizontal panel). The submotifs are encoded as fuzzy sets based on their score distributions. The distances are approximated by their distributions (*close*, *medium*, and *far* distances from left to right panels), and also encoded by fuzzy sets. Both antecedents are combined a fuzzy *AND* operator (*i.e.*, *product*). The consequent of a rule classifies PhoP BSs in the unit interval as a function of the antecedents (1: high, 0: low). Rules are activated concurrently, when they exceed each rule-specific threshold. The isobars show the degree of membership of the training set to the rules (white: none; red: high; blue: low). **B**) Synthesis of the PhoP BSs recognized by the most representative IF-THEN rules that distinguish *E. coli* K-12 (green) from *S. typhimurium* (red) genomes. The percentage of BSs recognized by each rule in each genome is represented by the height of the bars. Submotifs were compressed into their most general families (*i.e.*, S01, S05, and S08 & S09) for simplicity. Three subsets of rules (*i.e.*, S01 and *close*, S08 & S09 and *close*; and S08 & S09 and *medium*) are similarly distributed in both genomes (grey boxes). Specific rules in *E. coli* (*i.e.*, S05 and *close*, green boxes), and in *Salmonella* (S01 and *medium*; S05 and *far*; and S8 & S9 and *far*, red boxes) were also identified. **C**) Same as in **B**) but applying IF-THEN rules in *Y. pestis*. No rules were identified for the S05 family of submotifs, as well as for *far* distances.

We examined the distances between the PhoP BSs and the RNAP BSs within a region from −90 bp upstream and 10 bp downstream of the transcription start site (TSS). We distinguished three sets of distances: *close*, *medium* and *far* from the TSS (Figure 9). The location of the *close* set peaks at −34 bp from the TSS, the *medium* set peaks at −44 bp from the TSS, and the *far* set peaks at −68 bp from the TSS. Then, we incorporated the distances between the PhoP and the RNAP BSs to the single motif classifier by using fuzzy AND/OR IF-THEN rules. These rules improved SCC by 21% (*i.e.*, 0.63 vs 0.83) for recognizing PhoP BSs compared to the single motif model (Figure S7). Again, the use of submotifs instead of a single motif resulted in a multi-classifier employing 24 of the 36 possible rules (Figure 9A; the optimization of the number of rules was analogous to that performed with the submotifs), improved SCC by 45% (*i.e.*, 0.63 vs. 0.91). This improvement was also seen when analyzing other regulators like CRP (See Text S5, Tables S9, S10, and Figure S8).

Three of the associations between submotifs and distances (*i.e.*, IF-THEN rules) are shared by the *E. coli* and *Salmonella* genomes (*i.e.*, S01 and *close*, S08 & S09 and *close*; and S08 & S09 and *medium* (Figure 9A)). We did identify one subset of rules that is *E. coli* specific (*i.e.*, S05 and *close*) and three subsets of rules that are *Salmonella* specific (S01 and *medium*; S05 and *far*; and S08 & S09 and *far*) (Figure 9B). These results suggest that the distance between PhoP and the RNAP provides insights into species-specific promoter architectures in closely-related genomes.

We extended our analysis by comparing the distances between PhoP BSs and the RNAP BSs in the more distantly-related *Yersinia* genomes. In addition to the lack of the S05 family of submotifs, we found that *Y. pestis* does not have PhoP BSs at *far* distances from the RNAP BS (Figure 9C). (These were genuine sites and inferred distances, as was validated experimentally by S1 mapping analysis [58] (Figure 8B)). Interestingly, the PhoP BS in the *Salmonella* xenolog *mgtC* promoter [58] was located closer to the RNAP in *Yersinia* than in it's *far* position in the *Salmonella* genome. Moreover, the PhoP BS in the *Salmonella mgtC* promoter is in the reverse orientation, located at half-integral turns of the DNA helix from the −10 region of the RNAP, whereas this promoter in *Yersinia* is in the direct orientation and at an integral turn of the helix from the −10 region of the RNAP. Overall, the identified rules encode both similar and different organizations of *cis*-elements, reflecting promoter architectures that remain conserved or that change during evolution [58].

## Discussion

### Distinguishing properties of the *D&C* method

We have described a flexible computational framework that improves the recognition of functional BS, differentiating them from a background of variable DNA sequences that do not play a direct role in gene regulation. The proposed method partitions BSs sequences into families of submotifs by employing a hierarchical, possibilistic clustering approach, extracting maximal information from the typically short BS sequences. It encodes submotifs into PWMs employing any of several available methods. Then, it integrates the submotifs into a multi-classifier optimized by multi-objective constraints, including accuracy and complexity.

We showed that the multi-classifier outperforms single cluster motif methods in identifying PhoP BSs, independent of the metrics used to characterize the performance [1]. Sensitivity analysis of the method revealed that the approach is robust, with minimum dependence on the method-specific parameters. The performance of the proposed framework can be further improved by the incorporation of other *cis*-acting features, such as the distance of the regulator BS from the RNAP BS. The existence of rules that associate specific submotifs with discrete distances to the RNAP BS suggests that these features are not independently organized in the promoter. This should not be surprising, given that a TF must interact with the RNAP to regulate gene expression. Thus, a comprehensive understanding of the regulatory elements governing transcription initiation would treat them together.

### Biological significance of the results provided by the *D&C* method

In addition to the computational utility of the submotifs used in a multi-classifier to accurately identify PhoP and other regulators BSs, we showed that the submotif approach can complement experimental assays in genome-wide analyses. (It helped identify PhoP BSs that had a detectable effect on transcription, even though the ChIP-chip technique gave negative results). Our approach was devoted to solve, at least in part, the previously reported incongruence between experimental and computational recognition of TF BSs [8], [35]. This was done by using families of submotifs, in contrast to the single consensus motif, thereby increasing the sensitivity for the BSs without losing specificity.

We also demonstrated that the families of submotifs add a novel component to characterize the evolution of the PhoP regulon. This evolution depends at least on the co-evolution of the target genes (*i.e.*, orthologs); the changes in the regulatory protein; and the BSs used by the protein to bind its target promoters. Despite the proposal that differences among BS motifs are the major causes of divergence between species [29], we have demonstrated by use of a two-way analysis of orthologous genes regulated by PhoP within gamma/enterobacteria that this is not universally the case: we observed that families of submotifs disappeared in distantly-related species like *Yersinia*, and that this loss is due to their high rate of evolution. In contrast, we observed that families of submotifs present in most of the gamma/enterobacteria analyzed harbor a significantly lower rate of evolution. We also found that some individual submotifs have a sporadic occurrence even among closely-related species/strains, which suggests that their rate of evolution could be partially obscured by frequent and different patterns of horizontal gene acquisition in these species [59]. Thus, the binding motifs are not the major cause of divergence between species in the studied system, but rather, the gain and loss of groups of genes and changes in the promoter architecture through evolutionary turnover events represent a significant source of inter-species variation.

We uncovered at least three different promoter architectures characterized by the distance between TF BSs and the RNAP. Most transcription factors activate transcription by making contact with either the α subunit (specifically, its C-terminal domain [CTD]) or the σ^70^ subunit (the most commonly used σ factor) of RNAP, both of which can bind DNA [60]. The location of the *close* set of promoters, a characteristic position of Class II promoters [10], display PhoP BSs completely overlapping the −35 region. This configuration is found in promoters lacking sequences with good matches to the −35 region, which are typically bound by a particular σ^70^ subdomain [10], [58], as has been reported for promoters activated by the regulatory proteins PhoB and VanR [61], [62]. These promoters primarily display PhoP boxes of the S01 family of motifs, located in a direct orientation with respect to the RNAP binding site.

The *medium* set of promoters locates the PhoP BS slightly upstream of −35 region. This small difference, often ignored in traditional phylogenetic footprinting approaches [12], may suggest a different promoter architecture that might correspond to a distinct regulatory mechanism [8]. For example, the *crcA* and the *pagP* genes of *E. coli* and *Salmonella*, respectively, are orthologous genes encoding proteins that are 84% identical [63]. However, the PhoP BS at the *crcA* promoter is of the S10 submotif and is located at 32 bp from the TSS [42], while the PhoP BS at the *pagP* promoter is of the S03 and is located 44 bp from the TSS.

The *far* set of promoters often corresponds to Class I promoters [10]. The PhoP protein appears to interact with the α-CTD in these promoters. The PhoP box in these promoters does not overlap with the RNAP binding site, and the α-CTD subunit of RNAP is required to promote transcription of the *pagC* gene *in vitro* [58]. These promoters often display PhoP boxes of the S05 family of motifs, which are primarily located in the reverse orientation with respect to the RNAP BS. The overrepresentation of this promoter architecture in *Salmonella* suggests that it is not an arbitrary association of *cis*-acting elements, but a reflection of the fact that most of these genes were horizontally-acquired in this genome (Hypergeometric test, *p-value*<0.01). These results suggest that the promoter architecture is a key feature that can be used to differentiate between species-specific gene regulation.

Overall, we can conclude that the PhoP protein recognizes a constrained set of sequences, which are well characterized by the families of submotifs presented in this work, and that these submotifs are not fortuitous computational constructs. The evolutionary dynamic of the submotifs, associated with turnover of both coding and/or non-coding sequences (*e.g.*, horizontally-acquired genome regions), supports the biological significance of the submotifs, and thus, provides a model of target gene evolution based on their BSs.

### Perspectives of the *D&C* approach

Our findings argue that understanding a cell's behavior in terms of differential expression of genes controlled by a TF requires a detailed analysis of the *cis*-acting promoter features. This is even more evident in complex scenarios, such as those of eukaryotic regulatory systems [29], where TF BSs are even shorter than those found in prokaryotes, located in wider promoter regions, and often require several TFs to activate target genes [50]. The strategy of deconstruction and re-synthesis presented here may help to tackle the diversity inherently found in these regulatory systems. We believe that by considering multiple models of the *cis*-acting elements (as opposed to the relying on a single consensus) it will be possible to detect and uncover similarities, as well as subtle differences between regulatory targets, providing a greater understanding of co-regulated promoter's behaviors.

## Materials and Methods

### Divide: Clustering TFBS sequences

#### Hierarchical clustering [64]

We transform nucleotides from BS sequences into dummy variables [65] and calculate the Euclidean distance matrix to create a dendrogram by employing the single linkage method (*i.e.*, nearest-neighbor). We use the inconsistency index implemented in the Statistic Toolbox of Matlab (V6.0) to detect the clusters that maximize the similarity.

#### Subtractive clustering [30]

This method iteratively selects submotifs that exhibit the highest density of sequences recovered by a PWM tool (*e.g.*, Consensus, MEME, AlignAce). The retrieved true positives sequences (*i.e.*, those above a threshold optimized by the SCC/CC measures) are removed from the training set to conform a cluster. This process is exhausted while the used measure is above 0.5.

#### Hierarchical possibilistic clustering

We use the Xie-Beni validity index (see below) to learn the number of clusters (*n*) that produce the optimal partition of the PhoP BSs dataset. Because there are more than one optimal partition of the dataset (*m*), we select all of their corresponding number of cluster 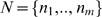. We apply the possibilistic fuzzy *c*-means algorithm (see below), initializing the number of clusters for each 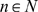, and generating 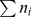 clusters. We organize the resulting clusters into a hierarchy by applying the hypergeometric test (see below) as a coincidence index among clusters [51].

#### Possibilistic fuzzy c-means

We transform nucleotides from BS sequences into dummy variables [65] and then apply the following algorithm [28], [64]: (i) Initialize the clustering partition 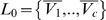, where 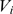 is a cluster and 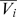 is its centroid; (ii) while (*s*<*S* and 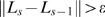), where *S* is the maximum number of iterations; (iii) calculate the membership 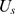 of each observation 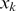 to each cluster 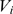 in 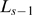 as 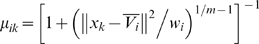, 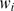 is the “bandwidth” of the fuzzy set, initialized as 1; *m* is the degree of fuzzification which is initialized as 2; (iv) update 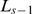 to 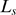 with 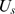 and 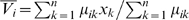; (v) iterate. (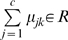, and it is not constrained to equal 1 [32]).

#### Xie-Beni validity index [28]

The minimization of this index through different number of clusters (*i.e.*, 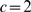 to 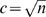, where *c* is the number of clusters and *n* the number of observations) detects compact representations of Fuzzy c-means partitions:
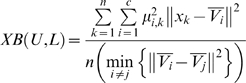
(1)


#### Hyergeometic test [51]

The coincidence between clusters of BSs is evaluated by using the hypergeometric distribution that gives the chance probability (*i.e.*, probability of intersection PI) of observing at least *p* candidates BSs from a cluster 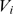 within another cluster 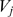 of size *n*:
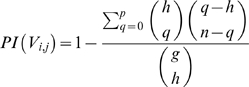
(2)where *h* is the total number of elements within 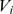, and *g* is the total number of BSs, such that the lower the *PI* the better the association.

### Combine: Voting multi-classifier

Each submotif is encoded as PWM that classifies a query sequence as positive TFBS if the corresponding score is above a threshold learned by GA (see below). We use a single voting strategy, where all PWMs vote, and one positive classification is sufficient to predict a TFBS. This process is analogous to a hierarchical Naïve Bayes [56]: 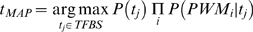 where 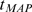 (maximumposterior probability) denotes the target output value of the Naïve Bayes classifier; 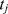 corresponds to the TFBS class; and 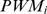 is by itself a Naïve Bayes classifier [14].

### Optimize: Genetic algorithms (GA)

We implemented a method that optimizes the multi-classifier [37] using GA to learn thresholds for the *m* PWMs composing it. Each allele in the chromosome is implemented as a pair, where the first element represents the presence/absence of a submotif (*i.e.*, >0.5 or <0.5), and the second element is its threshold defined in the unit interval [66]. We employ the “Max Min arithmetical” crossover [67], which given two solutions 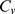 and 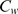 to be crossed generates four offspring and picks the one with best fitness:
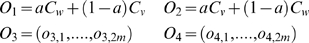
(3)where 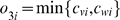, 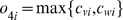, and 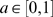 is chosen randomly following an uniform distribution. The mutation operator in the first element of the pair switches from presence to absence of a submotif, or viceversa, with probabilities *p* = 0.05 and *p* = 0.005, respectively. The mutation operator in the second element of the pair increases or decreases the corresponding threshold up to 10% of its value. The fitness function evaluates the CC or SCC (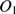) (see below) measures for the set of submotifs encoded in each chromosome. The multi-objective implementation includes the complexity of the model (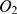) (*i.e.*, number of used submotifs/total number of submotifs) into the fitness function:
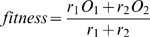
(4)where 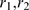 are user-dependent parameters, which are simply initialized as 1 if no preference exist among objectives [33], [66]. We use the Matlab implementation of GA (*i.e.*, Genetic Algorithm and Direct Search toolbox, Version 2.1), with the default values for the remaining parameters. Other optimization methods can also be used with lower performance (*i.e.*, Matlab Optimization Toolbox V3.1.1). (See Text S3, Figure S4, and Figure S9 for detailed analysis of results of the optimization process).

#### Correlation coefficient (CC)

This measure is based on the Pearson product-moment coefficient of correlation that indicates the relation between predicted and observed values, and is suitable for balanced datasets:
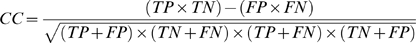
(5)where *P = positive*, *N = negative*, *T = true and F = false* [68].

#### Standardized correlation coefficient (SCC)

The standardized version considers the magnitude of the positive and negative examples, resulting in an appropriate choice where the dataset presents an unbalanced number of positive and negative examples. It extends the CC (equation (5)) replacing its parameters:
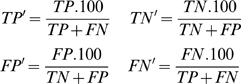
(6)


### Fuse: Identifying and executing fuzzy IF-THEN rules

We combine different sources of knowledge, such as TFBS motifs and their location relative to the RNAP BSs, by using a common framework of fuzzy IF-THEN rules [66], [69]. These rules provide accurate predictive results, and more importantly, are easily interpretable [66]. The fusion strategy consist of three phases: *i*) Encoding distinct sources of knowledge into fuzzy sets [69]; *ii*) Generating rules by connecting fuzzy sets from *i*) as their antecedents, and formulating their corresponding consequents; and *iii*) Optimizing the set of rules identified in *ii*), eliminating their redundancy, and thus improving their classification power.

#### Encoding PWMs as fuzzy sets

We normalize the scores provided by a PWM into the unit interval, where 1 indicates a perfect match and 0 a mismatch. These scores can be interpreted as the memberships to a fuzzy set, where the PWM is its centroid. Formally, a dataset of sequences corresponding to BSs 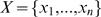 can be described by their memberships to a fuzzy set 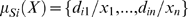, where 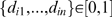 and represent the degree of matching between an observation of the dataset and a fuzzy set. Thus, a family of *n* submotifs can be represented by a collection of fuzzy sets 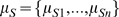.

**Encoding distances between TF and RNAP BSs as fuzzy sets:** Fuzzy sets can be viewed as an approximation of a data distribution, where the degree of matching between an observation and those sets is calculated in the [0,1] scale by using different membership functions [69]. Therefore, we uncover the distribution of the distances between TFBS and RNAP by representing them by as histograms. Then, we projected the histograms onto the variable domains by simple regression and minimum squared methods [65], [70]. The degree of matching between the set of distances 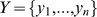 is calculated by using a triangular membership function:
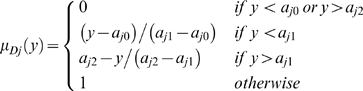
(7)where 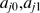 and 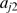 parameters are learned from the projection of the data into the variable domain.

This process is analogous to fitting a distribution, and assigning probability values to the observations based on a density function [65], [70]. The learned distributions of distances are represented as *m* fuzzy sets 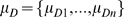, and implemented using the Matlab Fuzzy Logic Toolbox version V2.2.5.

#### Identifying fuzzy IF-THEN rules

A fuzzy rule is defined as a conditional statement where both the antecedent and consequent are fuzzy variables [28], [69]. The antecedent of a rule is a relation among the fuzzy sets characterizing the studied variables, here termed “submotifs” and “distances from a TF and RNAP BSs ”. To identify significant fuzzy rules from the dataset we calculated the Cartesian product among the fuzzy sets describing the former variables 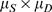; evaluated the probability of intersection of each pair by using the hypergeometric test (equation (2)); and selected those related pairs that showed a *p-value*<0.0001. The consequent of a rule is the class *C* of the TFBS. Finally, a fuzzy IF-THEN rule is defined as 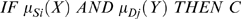.

The inference process consists of determining a classification value from the complete set of significant fuzzy rules. One rule is activated to a certain degree *th* as a result of the conjunction of its antecedent variables. Here we use the *product* as an AND operator [28], [69]. Then, the degrees of activation of the rules are combined using a defuzzify-then-combine strategy [71] based on the *maximum* operator [28], [69]. Thus a sequence is predicted to be a TFBS if at least one of these rules is satisfied. Again, this process can be implemented as a hierarchical Bayesian classifier (see above), but with less interpretability.

#### Optimizing fuzzy IF-THEN rules

We employed the GA described above to optimize the fuzzy rules. Each allele in the chromosome is implemented as a pair, where the first element represents the presence/absence of a rule (*i.e.*, >0.5 or <0.5), and the second element is the threshold of the rule (*th*).

### Availability

The programs, scripts and datasets used in this work are available at gps-tools2.wustl.edu, or can be requested to the authors.

### Rate of evolution for BSs

We measured the rate of evolution in substitutions per site, where site refers to a single nucleotide position in the BS of a TF. To do so, and following procedures described in [18], [19], we employed the model of Halpern and Bruno (HB) [57] that gives the rate of evolution *R* of base *a* to base *b* at position *p* as:
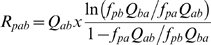
(8)where *Q* is the position-independent mutation matrix, and *f* is the PWM corresponding to a submotif. To estimate the evolutionary distance (rate×time) measured in substitutions, we assume that the time for all sites within one species are the same. Consequently, we can infer differences in rates based on differences in distances [19]. We therefore set background non-coding evolutionary (distance) model equal to *Q*, and estimated it employing the HYPHY package [72]. Thus, we learned the background HKY85 model on a set of aligned 1000 random sequences of 19 bp belonging to the non-coding regions of *Salmonella*. We also learned this model using the same number and length of random sequences from non-coding regions corresponding to horizontally-acquired genes (*i.e.*, AT rich regions). To predict the expected distance *K* at each position we used 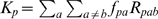 for HB and 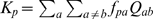 for HKY85.

### Gamma/enterobacteria orthologs

Given a gene or a list of genes from a query organism sequences (*E. coli*, *Salmonella* and *Y. pestis*), and a reference taxon (gamma/enterobacteria) we obtain the orthologous of the query gene(s) in all the organisms belonging to the reference taxon [43] (http://rsat.ulb.ac.be/rsat/get-orthologs_form.cgi).

### RNA isolation and expression microarray analysis

*S. typhimurium* strain harboring a chromosomally-encoded HA-tagged *phoP* gene, which was constructed previously in our lab. [73], was grown at 37°C in N-minimal medium [74] buffered in 50 mM Bis-Tris, pH 7.7, supplemented with 0.1% casamino acids, 38 mM glycerol and 50 µM or 10 mM MgCl_2_. After overnight culture in defined medium containing 10 mM MgSO_4_, *S. typhimurium* cells were grown to exponential phase (A_600_∼0.4) in same medium. Then, 10ml cells were washed with Mg^2+^-free medium and grown 10 ml of medium containing 50 µM MgSO_4_ with vigorous shaking for 1 hour. 5 ml of cell culture were collected, mixed with RNAprotect Bacteria Reagent (Qiagen) and used to prepare total RNA using RNeasy Mini Kit (Qiagen). RNA samples were treated with Turbo DNA-free DNase (Ambion) and re-purified with the RNeasy Mini Kit. *S. typhimurium* tiling arrays were manufactured by NimbleGen Systems Inc (Madison). RNA labeling, array hybridization and data extraction were carried out according to standard operating procedures by NimbleGen Systems Inc (Madison).

### Chromatin immunoprecipitation (ChIP) assay

Cells were grown in N-minimal medium containing 10 mM MgCl_2_ to OD_600_∼0.6. 10 ml of cell culture were washed with Mg^2+^-free medium and inoculated into 20 ml of fresh medium containing 50 µM MgCl_2_. Cells were then grown with vigorous shaking at 37°C for 20 min. ChIP assays were carried out as described [73] with the following modifications: PhoP-HA-crosslinked DNA was immunoprecipitated with anti-HA H6908 (Sigma) and the latter captured with Protein G sepharose (GE Healthcare). After reversal of crosslinking, the immunoprecipitated (IP) and input DNA were purified using QIAquick columns (Qiagen) following manufacturer's instructions. To generate enough material for hybridization, two rounds of genome amplification were carried out with the IP and input DNA samples as described [75] using GenomePlex Complete Whole Genome Amplification Kit (Sigma). We performed three independent ChIP assays.

### Microarray and ChIP-chip analysis

The experiments were conducted in triplicate to determine the error due to technical aspects of the process. Systematic error [76] was treated by a the Moderated t-Test [77], which is similar to the Student's t-Test in that it is used to compare the means of probe expression values for replicates for a given gene. The Student's t-Test calculates variance from the data that is available for each gene, while the Moderated t-Test uses information from all of the selected probes to calculate variance.

To correct for multiple test (*i.e.*, false positives within a large dataset), we used the Benjamini Hochberg method [78], which is not as conservative as the Bonferroni approach. This method aims to reduce what is called the False Discovery Rate (FDR) and is used when the objective is to reduce the number of false positives and to increase the chances of identifying all the differentially expressed genes. In this method, the *p-values* are first sorted and ranked. The smallest value gets rank 1, the second rank 2, and the largest gets rank N. Then, each *p-value* is multiplied by N and divided by its assigned rank to give the adjusted *p-values*. In order to restrict the false discovery rate to 0.05, all the probes with adjusted *p-values* less than 0.05 are selected.

Probes that exhibit differential expression all through the six experiments were selected. Overall, 1463, 1998, 2319 probes were identified at 99%, 95%, and 90% of confidence, respectively; and 2285 and 1273 show 4 and 8 fold changes, respectively. Altogether, 1195, 1263, 1268 probes at 99%, 95% and 90% confidence exhibit 8-fold changes; and 1148, 1930 and 2072 99%, 95% and 90% confidence exhibit 4-fold changes. The significant expressed ORFs were identified by collating the extracted probe locations with the *S. typhimurium* genome.

The ChIP microarray (ChIP-chip) data were analyzed as follows. Signal intensity data were extracted from the scanned images of each array using NimbleScan. A scaled Log_2_-ratio of the co-hybridized input and IP samples was calculated for each tile on the array. This ratio was computed to center the ratio data around zero. Scaling was performed by subtracting the bi-weight mean for the log2-ratio values for all tiles on the array from each Log_2_-ratio values. Peaks were detected by searching for 4 or more tiles whose signals were above a cutoff value (ranging from 90% to 15% of a hypothetical maximum defined as the mean + 6 standard deviations) using 500 bp sliding window. The ratio data was then randomized 20 times to evaluate the false positive probability. Each peak was then assigned a false discovery rate (FDR) score based on the randomization.

## Supporting Information

Text S1 Different clustering methods employed for the “divide” phase recover different position-dependent conserved patterns.
(0.07 MB DOC)

Text S2 A multi-classifier based on submotifs outperforms the single motif prediction of CRP BSs.
(0.05 MB DOC)

Text S3 Multi-objective optimization and performance evaluation of the multi-classifier.
(0.08 MB DOC)

Text S4 Results that require further experiments to validate the functionality of some of vague BSs.
(0.07 MB DOC)

Text S5 Combining cis-features and submotifs into a multi-classifier that detects CRP BSs.
(0.07 MB DOC)

Table S1 PhoP classifiers obtained by employing different clustering methods. CC: Correlation Coefficient; SCC: Standardized Correlation Coefficient.
(0.04 MB PDF)

Table S2 Performance of the CRP Single Motif classifier P: positive, N: negative, T: true and F: false; CC: Correlation Coefficient; SCC: Standardized Correlation Coefficient; SP/SN stands for specificity and sensitivity, respectively.
(0.04 MB PDF)

Table S3 CRP classifiers obtained by employing different clustering methods. (*) CC: Correlation Coeffient; SCC: Standardized Correlation Coefficient.
(0.04 MB PDF)

Table S4 10-fold cross-validation for the Divide & Conquer approach applied to the PhoP BSs. (*) CC: Correlation Coeffient; SCC: Standardized Correlation Coefficient.
(0.22 MB PDF)

Table S5 PhoP submotifs-leave-one-submotif out crossvalidation. First line of each cell shows the number of BSs recoverd by PWMs from other submotifs; and the second line the p-value calculated by the hypergeometric test.
(0.29 MB PDF)

Table S6 Genome-wide analysis of *Salmonella* using PhoP submotifs.
(0.13 MB PDF)

Table S7 Genome-wide analysis of the *S. typhimurium* sequences using PhoP submotifs, gene expression and promoter occupancy of the PhoP protein.
(0.04 MB PDF)

Table S8 Genome-wide analysis of *Y. pestis* using PhoP submotifs
(0.35 MB PDF)

Table S9 CRP classifier using single motif and distances between CRP and RNAP BSs. (*) CC: Correlation Coeffient; SCC: Standardized Correlation Coefficient.
(0.14 MB PDF)

Table S10 CRP classifier using submotifs and distances between CRP and RNAP BSs. (*) CC: Correlation Coeffient; SCC: Standardized Correlation Coefficient.
(0.14 MB PDF)

Figure S1 Intrinsic properties of the clustering algorithms recover distinct CRP Submotifs. A) The Subtractive Submotif 1 (SS1) groups 58 BSs that are assigned to two disjoint submotifs by the hierarchical algorithm: HS1, which has a total of 47 BSs; and HS2, which has a total of 32 BSs. Although there is a high degree of inclusion between SS1 and HS1 (p-value = 8.40E-04) and between SS1 and HS2 (p-value = 6.10E-03), the submotifs exhibit different patterns. SS1 encodes a general pattern that shows a balanced conservation between both tandems. In contrast the HS1 and HS2 exhibit more specific patterns with higher conservation of the second and first tandems respectively. C) Hierarchical possibilistic submotif 8 (HPS8) includes a mixture of 15 sequences from HS1 (p-value = 0.59), 3 sequences from HS6 (p-value = 0.59), and 3 sequences from HS7 (p-value = 0.53). Although the intersection of HPS8 cluster to the above clusters is not significant, its corresponding patterns shares with HS1 the second tandem, with HS6 the “GTGA” sequence; and with HS7 the “TGT-A” sequence.
(0.07 MB PDF)

Figure S2 PhoP submotifs learned by the subtractive clustering method. Three PhoP submotifs generated by the subtractive clustering. Their incorporation into a multi-classifier improved SCC by 33% (i.e., 0.73 vs. 0.547) and CC by 25% (i.e., 0.822 vs. 0.653) with respect to the single motifs. Visually, the patterns revealed slight differences among them, resulting in a decreasing level of nucleotide conservation (i.e., 13.84; 9.72 and 6.76 information content, respectively). Only one significant (p-value = 0.002) coincidence exist among the submotifs generated by the subtractive and the hierarchical possibilistic clustering methods (i.e., 6 of the 11 BSs forming the SS1 submotif coincide with 6 of the 13 BSs forming the S03 submotif).
(0.06 MB PDF)

Figure S3 Information content of PhoP submotifs. The four most general submotifs detected by the hierarchical possibilistic method (orange) exhibit a higher information content than those learned by the subtractive (green) and the single motif method (dashed blue).
(0.06 MB PDF)

Figure S4 Comparison among of different clustering and PWM methods uncovering CRP submotifs. Sets of submotifs generated by different clustering (shapes) and PWM (colors) methods are evaluated by their complexity (Y axis) and their performance, where the SCC metric was displayed as 1-SCC across the X axis. We identified all optimal solutions lying in the Pareto optimal frontier [9]. This frontier is the collection of multi-objective optima in the sense that its members are not worse than (i.e., dominated by) other solutions in any of the objectives being considered.
(0.07 MB PDF)

Figure S5 Optimal configurations of PhoP submotifs encoded into PWM using A) MEME and B) AlignACE. GA optimization was applied on the number and thresholds of submotifs (CF). The fitness function was calculated by either SCC or CC measurements (OO). Different selection pressures (SN) where used as initial constrains (See parameter in Materials and Methods). TP/TN and FP/FN stand for true/negative and positive/negative predicted values, respectively. #Sub indicates the number of submotifs effectively employed, columns S1 to S12 represent the submotifs organized as families. Dots at the columns (black: general submotif; white: specific submotif) indicate that the corresponding submotif was selected by the optimization process for that configuration (rows). Min Th. corresponds to the minimum learned threshold. SM shows the results obtained by the single motif.
(0.06 MB PDF)

Figure S6 Evolution of the PhoP protein. A) Tree indicating the phylogenetic relationship among the PhoP protein for members of gama/enterobacterias. B) Alignment of the PhoP DNA-binding domain.
(0.56 MB PDF)

Figure S7 IF-THEN rules encompassing PhoP single motif and distances between PhoP BSs and RNAP BSs in activated promoters. Rows correspond to IF-THEN rules. The antecedent of the rules is composed of a single motif (left panel) and the distances between PhoP BSs and RNAP BSs (middle panel). The single motif is encoded as a fuzzy set based on their score distributions. The distances are approximated by their distributions (close, medium, and far distances from left to right panels), and also encoded by fuzzy sets. Both antecedents are combined a fuzzy AND operator (i.e., product). The consequent of a rule classifies PhoP BSs in the unit interval as a function of the antecedents (1: high, 0: low). Rules are activated concurrently, when they exceed each rule-specific threshold. The isobars show the degree of membership of the training set to the rules (red: high; blue: low).
(0.06 MB PDF)

Figure S8 IF-THEN rules encompassing CRP submotifs and distances between CRP BSs and RNAP BSs. Rows correspond to IF-THEN rules. The antecedent of the rules is composed of submotifs (left panel) and the distances between PhoP BSs and RNAP BSs (middle panel). The submotifs are encoded as fuzzy sets based on their score distributions. The distances are approximated by their distributions (close, medium, and far distances from left to right panels), and also encoded by fuzzy sets. Both antecedents are combined a fuzzy AND operator (i.e., product). The consequent of a rule classifies PhoP BSs in the unit interval as a function of the antecedents (1: high, 0: low). Rules are activated concurrently, when they exceed each rule-specific threshold. The isobars show the degree of membership of the training set to the rules (red: high; blue: low).
(0.07 MB PDF)

Figure S9 ROC curves comparing the performance of classifiers based on submotifs and single motif. ROC curves corresponding to the single motif classifier (black line), and to multi-classifiers using all submotifs (red line), the most specific set of submotifs (blue line), and the most general set of submotifs (green line). The performance of the single motif is outperformed by all multi-classifiers using submotifs.
(0.03 MB PDF)
