# Supplementary material for: Defining the Plasticity of Transcription Factor Binding Sites by Deconstructing DNA Consensus Sequences: The PhoP-Binding Sites among Gamma/Enterobacteria
Source: PLoS Comput Biol. 2010 Jul 22;6(7):e1000862. doi: 10.1371/journal.pcbi.1000862 (PMC2908699; doi:10.1371/journal.pcbi.1000862)
Supplement: Text S1 — Different clustering methods employed for the “divide” phase recover different position-dependent conserved patterns. (0.07 MB DOC) [file pcbi.1000862.s001.doc]

**Defining** **the plasticity of transcription factor binding sites by deconstructing DNA consensus sequences**

**SUPPLEMENTAL TEXT S1: Different clustering methods employed for the “*divide*” phase recover different position-dependent conserved patterns**

We learned submotifs for binding sites of the cyclic AMP receptor protein (CRP), a well characterized master regulator of >100 genes in *Escherichia coli*. The hierarchical clustering method [1] subdivided the CRP BSs into 7 subsets (*i.e.*, hierarchical submotifs (HS)), whereas the subtracting clustering method [2] subdivided them into 5 subsets (*i.e.*, substrative submotifs (SS)). We found both coincidences and differences between the two clustering methods (Figure S1). For example, the SS5 and the HS3 clusters exhibited high levels of coincidence (*p-value* < 5.90E-04), and thus, represented the same submotif. This is also true for SS3 and HS4 (*p-value* < 2.60E-03), and for SS2 and HS7 (*p-value* < 3.70E-03). However, the SS1 cluster, describing 58 BSs, is split into two disjoint sets by the hierarchical method: the HS1 cluster, harboring 47 BSs (*p-value*=8.40E-04), and the HS2 cluster, with 31 BSs (*p-value* < 6.10E-03) (Figure S1A). These differences reflect distinct patterns encoded in the submotifs. For example, SS1 encodes a pattern that shows a balanced conservation between both CRP tandems. By contrast, the HS1 and HS2 are more specific patterns that individually reflect a well conserved second and first tandem repeats, respectively.

One of the major difficulties arised when clustering short sequences, as is the case of TFBSs, is that crisp clustering methods assign sequences to single but unstable clusters (*i.e.*, low bootstrap values). In other words, the clusters are highly overlapping because one sequence can belong to more than one group. To solve this problem, we introduced the hierarchical possibilistic clustering method [1,3], which is a variant of the classical fuzzy C-means algorithm, that encodes memberships to multiple clusters (see Materials and Methods). This approach (Figure S1) preserved the original well-defined clusters (*e.g.*, HS1 and HPS3, *p-value*< 4.64E-10; HS4 and HPS2, *p-value*<3.51E-14; and HS5 and HPS4, *p-value*<=4.28E-09). Yet, recovered more informative submotifs (Figure S1B) as measured by the information content (IC) [4] (*e.g.*, HPS8 (IC=13.91) includes sequences from HS1 (IC=10.68), HS6 (IC=10.22), and HS7 (IC=8.52)).

**REFERENCES**

1. Gasch AP, Eisen MB (2002) Exploring the conditional coregulation of yeast gene expression through fuzzy k-means clustering. Genome Biol 3: RESEARCH0059.

2. Hering JA, Innocent PR, Haris PI (2004) Beyond average protein secondary structure content prediction using FTIR spectroscopy. Appl Bioinformatics 3: 9-20.

3. Bezdek JC (1998) Pattern Analysis. In: Pedrycz W, Bonissone PP, Ruspini EH, editors. Handbook of Fuzzy Computation. Bristol: Institute of Physics. pp. F6.1.1-F6.6.20.

4. Hertz GZ, Stormo GD (1999) Identifying DNA and protein patterns with statistically significant alignments of multiple sequences. Bioinformatics 15: 563-577.
