# Supplementary material for: Defining the Plasticity of Transcription Factor Binding Sites by Deconstructing DNA Consensus Sequences: The PhoP-Binding Sites among Gamma/Enterobacteria
Source: PLoS Comput Biol. 2010 Jul 22;6(7):e1000862. doi: 10.1371/journal.pcbi.1000862 (PMC2908699; doi:10.1371/journal.pcbi.1000862)
Supplement: Text S2 — A multi-classifier based on submotifs outperforms the single motif prediction of CRP BSs. (0.05 MB DOC) [file pcbi.1000862.s002.doc]

**Defining** **the plasticity of transcription factor binding sites by deconstructing DNA consensus sequences**

**SUPPLEMENTAL TEXT S2: A multi-classifier based on submotifs outperforms the single motif prediction of CRP BSs**

The submotif approach provides a set of alternative and complementary models of DNA sequences describing TFBS. This requires a coordinating strategy to allow their usage as predictors when screening DNA sequences. We integrated these models into a voting multi-classifier [1], where each model votes above an individual threshold that reflects the similarity of a querying sequence with the corresponding submotif. This simple concept exploits the specialization of each submotif to recognize particular patterns. The strategy can result in an excessive number of overfitted models, each supported by few observations [2]. Moreover, the resulting redundancy can produce a poor classification performance [3]. Therefore, we designed a multi-objective genetic algorithm (GA) that optimizes thresholds for each model and appropriately integrates them into a cooperative classifier, which eventually constrains the influence of any redundant model.

We applied the *Divide & Conquer* approach to uncover the targets of the CRP protein, considering 148 CRP BSs as positive examples and 622 BS of other TFs as negatives examples in promoter sequences reported in the RegulonDB database [4] (Table S2). We found that the use of submotifs in a classifier increases 22.57% the SCC in average, obtaining an improvement up to 17% for AlignACE (*i.e.,* 0.692 vs. 0.592); 43.7% for MEME (*i.e.,* 0.682 vs. 0.475) and 22.69% for Consensus (*i.e.,* 0.734 vs. 0.598). This enhancement is mostly due to a substantial gain of sensitivity (27.08%), and a slightly increase of specificity (2.54%) (Table S3 and Figure S3). Comparable improvements were obtained optimizing CC (Table S3 and Figure S3).

The above results can be also evaluated by their complexity, when the number of submotifs is optimized by the GA. For example, the GA can build a multi-classifier employing only 5 of the original 8 submotifs obtaining an improvement of 13% of the SCC using Consensus PWMs. It can also reduce the usage of submotifs up to 2 representatives from the original 7, obtaining an improvement of 23% of the SCC using MEME PWMs. Indeed, it is able to construct a multi-classifier based on 5 submotifs outperforming the single motif by 14% utilizing AlignAce PWMs (Figure S4). This indicates that *D&C* improves the classification performance even when employing simplified models.

**REFERENCES**

1. Bauer E, Kohavi R (1999) An empirical comparison of voting classification algorithms: Bagging, boosting, and variants. Machine Learning 36: 105-139.

2. Ruspini EH, Zwir I (2002) Automated generation of qualitative representations of complex objects by hybrid soft-computing methods. In: Pal SK, Pal A, editors. Pattern recognition : from classical to modern approaches. New Jersey.: World Scientific. pp. 454-474.

3. Zwir I, Shin D, Kato A, Nishino K, Latifi T, et al. (2005) Dissecting the PhoP regulatory network of Escherichia coli and Salmonella enterica. Proc Natl Acad Sci U S A 102: 2862-2867.

4. Salgado H, Santos-Zavaleta A, Gama-Castro S, Millan-Zarate D, Diaz-Peredo E, et al. (2001) RegulonDB (version 3.2): transcriptional regulation and operon organization in Escherichia coli K-12. Nucleic Acids Res 29: 72-74.
