# Supplementary material for: Defining the Plasticity of Transcription Factor Binding Sites by Deconstructing DNA Consensus Sequences: The PhoP-Binding Sites among Gamma/Enterobacteria
Source: PLoS Comput Biol. 2010 Jul 22;6(7):e1000862. doi: 10.1371/journal.pcbi.1000862 (PMC2908699; doi:10.1371/journal.pcbi.1000862)
Supplement: Text S3 — Multi-objective optimization and performance evaluation of the multi-classifier. (0.08 MB DOC) [file pcbi.1000862.s003.doc]

**Defining** **the plasticity of transcription factor binding sites by deconstructing DNA consensus sequences**

**SUPPLEMENTAL TEXT S3: Multi-objective optimization and performance evaluation of the multi-classifier**

The sensitivity of the proposed multi-classifier is dictated by two types of parameters: the thresholds of the submotifs, and the number of submotifs considered. Here we show results of learning optimal configurations of these parameters by using a multi-objective optimization algorithm based on GA [1]. This approach optimizes both: the accuracy (*i.e*., CC or SCC, see below) and the complexity of the multi-classifier (*i.e.*, number of submotifs). ( See [2] for a review).

In addition, we provide a two-ways evaluation of the multi-classifier performance. In machine learning, the performance of an algorithm is evaluated by its bias and variance [3,4]. The term "bias" was introduced by Mitchell [5] to mean any basis for choosing one generalization (hypothesis) over another, other than strict consistency with the observed training instances. The bias of a learning algorithm (for a given learning problem and a fixed size m for training sets) is the persistent or systematic error that the learning algorithm is expected to make when trained on training sets of size m [3,4,6]. To evaluate the bias of the proposed multi-classifier we used the receiver operating characteristic analysis (ROC) [7]. The variance captures random variation in the algorithm from one training set S to another. This variation can result from variation in the training sample, from random noise in the training data. Methods based on voting such as the proposed multi-classifier can reduce variance [3]. To evaluate the variance of the proposed multi-classifier we performed a leave-one-submotif-out to analyze the extent of overlap among families of submotifs, and a cross-validation analysis to assess how the results will generalize to an independent data set.

*Measurements of performance.* We employ two measurements of performance: the CC (*i.e.*, Pearson product-moment correlation coefficient) [8,9] or its modified version SCC for unbalanced number of positive and negative examples. The number of TP examples consisting of the TPBSs for a given TF is always much lower than the TN consisting of all other TFBSs in a database [10]. Therefore, the SCC measurement balances the reduced set of TP, and as a consequence, is mostly affected by the Type I error [8] (*i.e.*, poor specificity). In contrast, the CC is more susceptible to the Type II error (*i.e.*, [poor sensitivity](http://en.wikipedia.org/wiki/Sensitivity_and_specificity)).

*Parameter estimation of the D&C multi-classifier: accuracy vs. complexity.* The selection of optimal thresholds is critical to obtain the best performance of the multi-classifier. Our method considers neither equal thresholds for all submotifs, nor a same cutoff for all branches in the hierarchical organization of the submotifs (*i.e.*, cutoff in a dendrogram). By contrast, the number of submotifs and their respective thresholds are learned by using a multi-objective optimization algorithm based on GA [1]. This process optimizes the accuracy (*i.e*., CC or SCC, see below), as well s the complexity of the multi-classifier (*i.e.*, number of submotifs), where that a subset of submotifs may perform (*i.e.,* cooperates) better than the full set for classification [11] when exhibit some degree of overlapping. The result of the optimization process is a Pareto optimal frontier of the non-dominated solutions –instead of a single solution— (*i.e.*, the set of solutions that are non-dominated, in the sense that there is no solution that is superior to the others in all objectives [1,12]) (Figure S4).

We found that complexity is partially correlated with accuracy (*i.e.,* *F*-statistic, *p-value*<0.023 for the CC and *p-value*<0.003 for the SCC measures) when using the Consensus method to encode the submotifs (Figure 4B). For example, using the consensus method, the 7^th^ configuration performs better than the 8^th^ configuration that employs 2 less submotifs. However, the 2^nd^ optimal configuration, including 9 of the 12 submotifs (*i.e.*, discarding the two general S05 and S09, and the specialized S11 submotifs), achieves identical SCC and CC to the exhaustive set of submotifs (Figure 4B). ). Similar results were obtained using MEME and AlignACE (Figure S5). In contrast to Consensus, there was no configuration that equaled the performance of the exhaustive set of submotifs (See Figure 4B vs. Figure S5). Notably, all configurations for all PWM methods preserve at least one member of each family of submotifs (Figure 4B and Figure S5). Nonetheless, all 8 configurations learned for the Consensus, MEME and AlignACE bettered the single motif performance, allowing the identification of at least 22, 25 and 14 additional BSs, that were otherwise undetected by the respective method(Figure 4B).

The multi-objective optimization process revealed that those results obtained by using the SCC measurement as a fitness function tend to have [poor specificity](http://en.wikipedia.org/wiki/Sensitivity_and_specificity). Therefore, the optimization process has to compensate such lack providing specific submotifs (Figure 4B)*.* In contrast those results obtained using the CC have [poor sensitivity](http://en.wikipedia.org/wiki/Sensitivity_and_specificity), and as expected, they were optimized by more general submotifs (Figure 4B). Overall, each optimized configuration conserves at least on submotif for each family (Figure 4B).

*Evaluating the bias of the D&C multi-classifier: specificity vs. sensitivity.* We evaluated the performance of the multi-classifier by a ROC curve, which is a graphical plot of the sensitivity vs. (1 − specificity) for a classifier system as its discrimination threshold is varied (Figure S9). This analysis provides tools to select possibly optimal models and to discard suboptimal ones independently from the class distribution. The best possible prediction method would approximate the upper left corner or coordinate of the ROC space, representing 100% sensitivity and 100% specificity. In contrast, a completely random classifier would give a point along a diagonal line (the so-called *line of no-discrimination*) from the left bottom to the top right corners. The ROC can also be represented equivalently by plotting the fraction of true positives (TPR = true positive rate) vs. the fraction of false positives (FPR = false positive rate). We evaluate the sensitivity of the classifier based on the thresholds of the submotifs. To do so, we apply the same incremental step and threshold for all submotifs. The obtained results suggest a considerable advantage of the submotifs with respect to the single motif model (Figure S9).

*Evaluating the variance of the D&C multi-classifier.* A leave-one-submotif-out analysis (Figure 4A) reported in the main text allowed us conclude that there is a low degree of overlap among families of submotifs, which indicates that each of these families is required to effectively model the entire dataset. Here, we quantify the sampling effect on the performance of the multi-classifier. Although the overfitting problem is intrinsically controlled by the optimization of two opposite objectives [1] (*i.e.* accuracy and complexity of the model), we also perform a 10-fold cross-validation to compute classification error (Crossvalind function, Bioinformatics Toolbox, Matlab 7.5 R2007B). In this kind of analysis, the training dataset is divided in 10 subsets, 9 of them used to train the multi-classifier and the remaining one employed as a test subset. This analysis reveals that the multi-classifier based on submotifs improved SCC by 36% in average (*i.e.*, 0.84 for the submotifs vs. 0.52 scored by the single motif) and CC 21% in an average (*i.e.*, 0.82 vs. 0.64) for the training set (Table S4). The evaluation of the test set reflected similar improvements: SCC by 32% (*i.e.*, 0.76 vs. 0.51) and CC by 21% (*i.e.*, 0.75 vs. 0.59). Notably, these reduced training sets still outperform the scores obtained by the single motif PWM trained with the entire collection of PhoP BSs (Figure 4B).

We observed that when the test subset mostly contains sequences from one submotif, the overall performance of the multi-classifier decreased. The same phenomena were observed for the single motif PWM. For example, when one test subtest includes all of the BSs of the S07 submotif and one of the S02 submotif (Figure 3) the performance of the multi-classifier substantially decreases (SCC=0.52 and CC=0.46), as well as the performance of the single motif PWM (SCC=0.30 and CC=0.40). This is because the classifiers cannot identify a complete novel class based on its previously observed examples (Figure 4A). Another 10-fold cross-validation experiment, this time performed with submotif balanced BSs (*i.e.*, BSs that conform the test subset are chosen to be uniformly distributed among all of the families of the submotifs) avoided this problem, and improved the multi-classifier as well as the single motif PWM performance.

**REFERENCES**

1. Deb K (2001) Multi-objective optimization using evolutionary algorithms. Chichester ; New York: John Wiley & Sons. xix, 497 p.

2. Ferri C, Hern J, ndez O, Modroiu R (2009) An experimental comparison of performance measures for classification. Elsevier Science Inc. pp. 27-38.

3. Friedman JH (1997) On Bias, Variance, 0/1\&mdash;Loss, and the Curse-of-Dimensionality. Kluwer Academic Publishers. pp. 55-77.

4. Dietterich TG (2000) An Experimental Comparison of Three Methods for Constructing Ensembles of Decision Trees: Bagging, Boosting, and Randomization. Kluwer Academic Publishers. pp. 139-157.

5. Mitchell TM (1997) Machine learning. New York: McGraw-Hill. xvii, 414 p.

6. Bauer E, Kohavi R (1999) An empirical comparison of voting classification algorithms: Bagging, boosting, and variants. Machine Learning 36: 105-139.

7. Wichard JD, Cammann H, Stephan C, Tolxdorff T (2008) Classification models for early detection of prostate cancer. J Biomed Biotechnol 2008: 218097.

8. Everitt B, Der G (1996) A handbook of statistical analysis using SAS. London: Chapman & Hall. 158 p.

9. Tompa M, Li N, Bailey TL, Church GM, De Moor B, et al. (2005) Assessing computational tools for the discovery of transcription factor binding sites. Nat Biotechnol 23: 137-144.

10. Salgado H, Gama-Castro S, Martinez-Antonio A, Diaz-Peredo E, Sanchez-Solano F, et al. (2004) RegulonDB (version 4.0): transcriptional regulation, operon organization and growth conditions in Escherichia coli K-12. Nucleic Acids Res 32: D303-306.

11. Cordon O, Herrera F, Zwir I (2002) Linguistic modeling by hierarchical systems of linguistic rules. Ieee Transactions on Fuzzy Systems 10: 2-20.

12. Ruspini EH, Zwir I (2002) Automated generation of qualitative representations of complex objects by hybrid soft-computing methods. In: Pal SK, Pal A, editors. Pattern recognition : from classical to modern approaches. New Jersey.: World Scientific. pp. 454-474.
