# Supplementary material for: Defining the Plasticity of Transcription Factor Binding Sites by Deconstructing DNA Consensus Sequences: The PhoP-Binding Sites among Gamma/Enterobacteria
Source: PLoS Comput Biol. 2010 Jul 22;6(7):e1000862. doi: 10.1371/journal.pcbi.1000862 (PMC2908699; doi:10.1371/journal.pcbi.1000862)
Supplement: Text S4 — Results that require further experiments to validate the functionality of some of vague BSs. (0.07 MB DOC) [file pcbi.1000862.s004.doc]

**Defining** **the plasticity of transcription factor binding sites by deconstructing DNA consensus sequences**

**SUPPLEMENTAL TEXT S4: Results that require further experiments to validate the functionality of some of vague BSs**

Here we provide a more detailed explanation of the results displayed in (Figure 5). We detected PhoP BSs in 34 of 37 genes displaying significant expression and ChIP scores (Figure 5). The remaining three genes have low peak scores (<0.38), which are only detectable in one of the three ChIP replicas, and their expression may reflect read-through transcription from an adjacent PhoP-activated gene. We did not detect BSs in 54 of the total 70 expressed genes without significant ChIP peaks, which suggest a pattern of indirect regulation (Figure 5, Table S6). 11 of these genes, organized in three operons, are known to be indirectly regulated by PhoP [1,2], and as expected, PhoP BS resembling submotifs were not detected. Similarly, PhoP BSs were not detected in 5 genes member of an operon whose first gene harbors a PhoP BS, and in 38 remaining genes. We detected BSs in 16 promoters of the 70 genes, where PhoP promotes transcription of its targets even though ChIP gives negative results (Figure 5). 7 of these genes have been proposed to be indirectly regulated by PhoP via another regulatory protein(s) (*e.g.*, *pagD*, *pagC*, *mgtC* and *phoN* [3]), but we found that PhoP directly regulates them [4]. The remaining 9 genes lacking a significant ChIP peak but harboring PhoP BSs resembling a submotif are predicted to be regulated in the same fashion.

We did not detect PhoP BSs in 57 genes lacking significant expression but harboring ChIP peaks (Figure 5), which may correspond to false positive ChIP results. We identified BSs in 14 genes divergently located with respect to significantly expressed genes harboring BSs used by PhoP to promote their transcription. Yet, we identified BSs in 3 other genes displaying low expression, but containing several probes expressed at higher levels than 2 folds (Figure 5). Further experiments will be required to validate the functionality of some of these sites having weak peaks, probe sets with low expression (~2 fold), and sequences resembling PhoP submotifs.

**REFERENCES**

1. Aguirre A, Cabeza ML, Spinelli SV, McClelland M, Garcia Vescovi E, et al. (2006) PhoP-induced genes within Salmonella pathogenicity island 1. J Bacteriol 188: 6889-6898.

2. Kato A, Latifi T, Groisman EA (2003) Closing the loop: the PmrA/PmrB two-component system negatively controls expression of its posttranscriptional activator PmrD. Proc Natl Acad Sci U S A 100: 4706-4711.

3. Lejona S, Aguirre A, Cabeza ML, Garcia Vescovi E, Soncini FC (2003) Molecular characterization of the Mg2+-responsive PhoP-PhoQ regulon in Salmonella enterica. J Bacteriol 185: 6287-6294.

4. Zwir I, Shin D, Kato A, Nishino K, Latifi T, et al. (2005) Dissecting the PhoP regulatory network of Escherichia coli and Salmonella enterica. Proc Natl Acad Sci U S A 102: 2862-2867.
