# Supplementary material for: Defining the Plasticity of Transcription Factor Binding Sites by Deconstructing DNA Consensus Sequences: The PhoP-Binding Sites among Gamma/Enterobacteria
Source: PLoS Comput Biol. 2010 Jul 22;6(7):e1000862. doi: 10.1371/journal.pcbi.1000862 (PMC2908699; doi:10.1371/journal.pcbi.1000862)
Supplement: Text S5 — Combining cis-features and submotifs into a multi-classifier that detects CRP BSs. (0.07 MB DOC) [file pcbi.1000862.s005.doc]

**Defining** **the plasticity of transcription factor binding sites by deconstructing DNA consensus sequences**

**SUPPLEMENTAL TEXT S5: Combining *cis*-features and submotifs into a multi-classifier that detects CRP BSs**

The interaction between a TF and the RNAP is often a critical determinant of gene expression [1]. Bacterial promoters regulated by activator proteins can be divided into two groups depending on the location of the TFBS, which determines the subunit of RNAP with which the activator interacts [2,3]. In Class I promoters, presumably long distances might leave space for the α-CTD interaction downstream the TFBS [4]. In contrast, in Class II promoters, short distances might constraint the α-CTD pattern to be located upstream the TFBS. Furthermore, gene expression data allows distinguishing between activated and repressed genes, which often correlate with the BS location relative to the transcription start site (TSS) [4].

To integrate the information about the distance between CRP and RNAP BSs into the proposed motif models, we compiled 136 reported CRP distances in RegulonDB [5] whose locations are between 110 bp upstream the TSS and 10 bp downstream. Then, we represented their distributions as histograms, and encoded these distributions into fuzzy sets [6-8]. We identified 3 sets (*i.e.,* *far*, *medium* and *close* distances) representing different activation distributions, and another 3 sets corresponding to repression distributions [9]. We also encoded a BS motif (PWM) into a fuzzy set by using the scores as measurements of similarity with a centroid [10]. Then, we connected the fuzzy sets corresponding to the motif and the distance distributions into IF-THEN rules by using the fuzzy logic AND-operator [11]. The consequents of these rules are two classes corresponding to CRP BSs and BSs from other TFs.

By applying the above process, we ended up with 6 rules (see Materials and Methods). The identified rules improved SCC by 15% using the single motif (Table S9, Figure S8). Interestingly, even if we consider the complete set of distances corresponding to all TFs reported for the *E. coli* genome [5], as opposed to those specific of CRP, the SCC is still improved by 12%. This suggests that this procedure can be extended to other TFs even if the specific distances from their BS to the TSS are not available.

Finally, we encoded the submotifs into fuzzy sets and integrated them with the distances into IF-THEN rules (See Materials and Methods). This resulted in an optimized multi-classifier that employed 35 of the 48 possible rules, improving SCC by 34.59% (*i.e.,* 0.753 vs. 0.56) with respect to the single motif (Table S10). Again, by using the distributions of distances learned from all TFs –instead of those for a specific TF— we ended up in a multi-classifier with 39 rules that still improved SCC by 33.50% (*i.e.*, 0.747 vs. 0.56). The integration of genomic *cis*-features and TFBS into a multi-classifier always outperformed those results obtained by a single feature. This argues against the assumption that promoter regulatory elements are independent features, and provides a more realistic representation of the genome information [12].

**REFERENCES**

1. Barnard A, Wolfe A, Busby S (2004) Regulation at complex bacterial promoters: how bacteria use different promoter organizations to produce different regulatory outcomes. Curr Opin Microbiol 7: 102-108.

2. Cox RS, 3rd, Surette MG, Elowitz MB (2007) Programming gene expression with combinatorial promoters. Mol Syst Biol 3: 145.

3. Elemento O, Slonim N, Tavazoie S (2007) A universal framework for regulatory element discovery across all genomes and data types. Mol Cell 28: 337-350.

4. Browning DF, Busby SJ (2004) The regulation of bacterial transcription initiation. Nat Rev Microbiol 2: 57-65.

5. Salgado H, Santos-Zavaleta A, Gama-Castro S, Millan-Zarate D, Diaz-Peredo E, et al. (2001) RegulonDB (version 3.2): transcriptional regulation and operon organization in Escherichia coli K-12. Nucleic Acids Res 29: 72-74.

6. Bezdek JC (1998) Pattern Analysis. In: Pedrycz W, Bonissone PP, Ruspini EH, editors. Handbook of Fuzzy Computation. Bristol: Institute of Physics. pp. F6.1.1-F6.6.20.

7. Gasch AP, Eisen MB (2002) Exploring the conditional coregulation of yeast gene expression through fuzzy k-means clustering. Genome Biol 3: RESEARCH0059.

8. Ruspini EH, Zwir I (2002) Automated generation of qualitative representations of complex objects by hybrid soft-computing methods. In: Pal SK, Pal A, editors. Pattern recognition : from classical to modern approaches. New Jersey.: World Scientific. pp. 454-474.

9. Collado-Vides J, Magasanik B, Gralla JD (1991) Control site location and transcriptional regulation in Escherichia coli. Microbiol Rev 55: 371-394.

10. Zwir I, Huang H, Groisman EA (2005) Analysis of differentially-regulated genes within a regulatory network by GPS genome navigation. Bioinformatics 21: 4073-4083.

11. Cordon O, Herrera F, Zwir I (2002) Linguistic modeling by hierarchical systems of linguistic rules. Ieee Transactions on Fuzzy Systems 10: 2-20.

12. Barash Y, Elidan, G., Friedman, N., Kaplan, T. Modeling Dependencies in Protein-DNA Binding Sites; 2003.
