# Supplementary material for: Defining the Plasticity of Transcription Factor Binding Sites by Deconstructing DNA Consensus Sequences: The PhoP-Binding Sites among Gamma/Enterobacteria
Source: PLoS Comput Biol. 2010 Jul 22;6(7):e1000862. doi: 10.1371/journal.pcbi.1000862 (PMC2908699; doi:10.1371/journal.pcbi.1000862)
Supplement: Table S1 — PhoP classifiers obtained by employing different clustering methods. CC: Correlation Coefficient; SCC: Standardized Correlation Coefficient. (0.04 MB PDF) [file pcbi.1000862.s006.pdf]

Table S1: PhoP classifiers obtained by employing different clustering methods.

	Consensus		MEME		AlignACE	
	CC	SCC	CC	SCC	CC	SCC
**Single Motif**	0.654	0.547	0.707	0.613	0.718	0.709
**Subtractive**	0.823	0.730	0.832	0.773	0.778	0.762
**Hierarchical**	0.859	0.799	0.843	0.806	0.790	0.785

(*) CC: Correlation Coeffient; SCC: Standardized Correlation Coefficient.
