# Supplementary material for: Defining the Plasticity of Transcription Factor Binding Sites by Deconstructing DNA Consensus Sequences: The PhoP-Binding Sites among Gamma/Enterobacteria
Source: PLoS Comput Biol. 2010 Jul 22;6(7):e1000862. doi: 10.1371/journal.pcbi.1000862 (PMC2908699; doi:10.1371/journal.pcbi.1000862)
Supplement: Table S2 — Performance of the CRP Single Motif classifier P: positive, N: negative, T: true and F: false; CC: Correlation Coefficient; SCC: Standardized Correlation Coefficient; SP/SN stands for specificity and sensitivity, respectively. (0.04 MB PDF) [file pcbi.1000862.s007.pdf]

Table S2: Performance of the CRP Single Motif classifier (*)

Method	TP	TN	FP	FN	SP	SN	CC	SCC
Consensus	86	586	56	62	0.913	0.581	0.502	0.524
MEME	60	620	22	88	0.966	0.405	0.475	0.448
AlignACE	97	596	46	51	0.928	0.655	0.592	0.607

(*) P: positive, N: negative, T: true and F: false; CC: Correlation Coefficient; SCC: Standardized Correlation Coefficient; SP/SN stands for specificity and sensitivity, respectively;
