# Supplementary material for: Defining the Plasticity of Transcription Factor Binding Sites by Deconstructing DNA Consensus Sequences: The PhoP-Binding Sites among Gamma/Enterobacteria
Source: PLoS Comput Biol. 2010 Jul 22;6(7):e1000862. doi: 10.1371/journal.pcbi.1000862 (PMC2908699; doi:10.1371/journal.pcbi.1000862)
Supplement: Table S3 — CRP classifiers obtained by employing different clustering methods. (*) CC: Correlation Coeffient; SCC: Standardized Correlation Coefficient. (0.04 MB PDF) [file pcbi.1000862.s008.pdf]

Table S3: CRP classifiers obtained by employing different clustering methods.

	Consensus		MEME		AlignACE	
	CC	SCC	CC	SCC	CC	SCC
**Single Motif**	0.516	0.598	0.475	0.525	0.592	0.592
**Subtractive**	0.642	0.683	0.599	0.592	0.659	0.662
**Hierarchical**	0.633	0.677	0.638	0.639	0.655	0.660
**Hierarchical Possibilistic**	0.706	0.734	0.677	0.682	0.654	0.665
