# Supplementary material for: Defining the Plasticity of Transcription Factor Binding Sites by Deconstructing DNA Consensus Sequences: The PhoP-Binding Sites among Gamma/Enterobacteria
Source: PLoS Comput Biol. 2010 Jul 22;6(7):e1000862. doi: 10.1371/journal.pcbi.1000862 (PMC2908699; doi:10.1371/journal.pcbi.1000862)
Supplement: Table S4 — 10-fold cross-validation for the Divide & Conquer approach applied to the PhoP BSs. (*) CC: Correlation Coeffient; SCC: Standardized Correlation Coefficient. (0.22 MB PDF) [file pcbi.1000862.s009.pdf]

Table S4. 10-fold cross-validation for the Divide & Conquer approach applied to the PhoP BSs

		Training		Test	
		CC	SCC	CC	SCC
Equally distributed	Single motif	0.648	0.542	0.595	0.516
	Submotifs	0.824	0.847	0.755	0.761
Fold capturing S07	Single motif	0.741	0.643	0.396	0.302
	Submotifs	0.860	0.855	0.461	0.520
Balanced	Single motif	0.648	0.542	0.661	0.574
	Submotifs	0.820	0.846	0.788	0.787
