# Supplementary material for: Defining the Plasticity of Transcription Factor Binding Sites by Deconstructing DNA Consensus Sequences: The PhoP-Binding Sites among Gamma/Enterobacteria
Source: PLoS Comput Biol. 2010 Jul 22;6(7):e1000862. doi: 10.1371/journal.pcbi.1000862 (PMC2908699; doi:10.1371/journal.pcbi.1000862)
Supplement: Table S5 — PhoP submotifs-leave-one-submotif out crossvalidation. First line of each cell shows the number of BSs recoverd by PWMs from other submotifs; and the second line the p-value calculated by the hypergeometric test. (0.29 MB PDF) [file pcbi.1000862.s010.pdf]

Table S5. PhoP submotifs-leave-one-submotif out crossvalidation*

		**S01**	**S02**	**S03**	**S04**	**S05**	**S06**	**S07**	**S08**	**S09**	**S10**	**S11**	**S12**
	**#BS**	**28**	**6**	**13**	**9**	**17**	**11**	**6**	**21**	**17**	**12**	**9**	**8**
**S01**	**28**	-	-	-	-	7<br>*0.48*	3<br>*0.87*	0<br>*1.00*	0<br>*1.00*	7<br>*0.48*	1<br>*0.99*	0<br>*1.00*	3<br>*0.65*
**S02**	**6**	-	-	2<br>*0.28*	1<br>*0.55*	0<br>*1.00*	1<br>*0.63*	0<br>*1.00*	0<br>*1.00*	0<br>*1.00*	0<br>*1.00*	0<br>*1.00*	1<br>*0.51*
**S03**	**13**	-	0<br>*1.00*	-	4<br>*0.05*	5<br>*0.14*	3<br>*0.30*	0<br>*1.00*	0<br>*1.00*	4<br>*0.34*	1<br>*0.92*	0<br>*1.00*	2<br>*0.43*
**S04**	**9**	-	0<br>*1.00*	4<br>*0.05*	-	1<br>*0.92*	0<br>*1.00*	0<br>*1.00*	0<br>*1.00*	2<br>*0.66*	0<br>*1.00*	0<br>*1.00*	0<br>*1.00*
**S05**	**17**	0<br>*1.00*	0<br>*1.00*	0<br>*1.00*	0<br>*1.00*	-	-	0<br>*1.00*	0<br>*1.00*	0<br>*1.00*	0<br>*1.00*	0<br>*1.00*	0<br>*1.00*
**S06**	**11**	0<br>*1.00*	0<br>*1.00*	0<br>*1.00*	0<br>*1.00*	-	-	1<br>*0.63*	0<br>*1.00*	0<br>*1.00*	0<br>*1.00*	0<br>*1.00*	0<br>*1.00*
**S07**	**6**	0<br>*1.00*	0<br>*1.00*	0<br>*1.00*	0<br>*1.00*	-	0<br>*1.00*	-	0<br>*1.00*	0<br>*1.00*	0<br>*1.00*	0<br>*1.00*	0<br>*1.00*
**S08**	**21**	0<br>*1.00*	0<br>*1.00*	0<br>*1.00*	0<br>*1.00*	0<br>*1.00*	0<br>*1.00*	0<br>*1.00*	-	-	-	-	0<br>*1.00*
**S09**	**17**	1<br>*0.99*	0<br>*1.00*	0<br>*1.00*	0<br>*1.00*	2<br>*0.95*	0<br>*1.00*	0<br>*1.00*	-	-	1<br>*0.97*	-	-
**S10**	**12**	0<br>*1.00*	0<br>*1.00*	0<br>*1.00*	0<br>*1.00*	0<br>*1.00*	0<br>*1.00*	0<br>*1.00*	-	-	-	1<br>*0.82*	0<br>*1.00*
**S11**	**9**	0<br>*1.00*	0<br>*1.00*	0<br>*1.00*	0<br>*1.00*	0<br>*1.00*	0<br>*1.00*	0<br>*1.00*	-	-	1<br>*0.82*	-	0<br>*1.00*
**S12**	**8**	1<br>*0.98*	0<br>*1.00*	0<br>*1.00*	0<br>*1.00*	2<br>*0.59*	0<br>*1.00*	0<br>*1.00*	0<br>*1.00*	-	0<br>*1.00*	0<br>*1.00*	-

* First line of each cell shows the number of BSs recoverd by PWMs from other submotifs; and the second line the *p-value* calculated by the hypergeometric test.
