# Supplementary material for: Defining the Plasticity of Transcription Factor Binding Sites by Deconstructing DNA Consensus Sequences: The PhoP-Binding Sites among Gamma/Enterobacteria
Source: PLoS Comput Biol. 2010 Jul 22;6(7):e1000862. doi: 10.1371/journal.pcbi.1000862 (PMC2908699; doi:10.1371/journal.pcbi.1000862)
Supplement: Table S6 — Genome-wide analysis of Salmonella using PhoP submotifs. (0.13 MB PDF) [file pcbi.1000862.s011.pdf]

Table S6. Genome-wide analysis of *Salmonella* using PhoP submotifs

Expression & binding

Gene	Operon	Code	FDR ChIP 1	FDR ChIP 2	FDR ChIP 3	Peak ChIP 1	Peak ChIP 2	Peak ChIP 3	Average	*Expression	Submotif	Score	Notes
mgtA	mgtA	STM4456	0.00E+00	0.00E+00	0.00E+00	2.22	1.88	2.54	2.21	>10	S01	0.98	
ybjX	ybjX	STM0940	0.00E+00	0.00E+00	0.00E+00	2.2	1.88	2.54	2.21	>10	S03	0.91	
virK	virK	STM2781	0.00E+00	0.00E+00	0.00E+00	1.82	1.88	2.54	2.08	>10	S11	0.92	
STM1829	STM1829	STM1829	0.00E+00	0.00E+00	1.00E-03	1.63	1.88	2.34	1.95	>10	S05	0.77	
rstA	rstA	STM1475	0.00E+00	0.00E+00	0.00E+00	1.41	1.88	2.45	1.91	>10	S02	0.84	
STM1254	STM1254	STM1254	0.00E+00	0.00E+00	0.00E+00	1.41	1.76	2.14	1.77	>10	S01	0.91	
STM1941	STM1941	STM1941	0.00E+00	0.00E+00	0.00E+00	1.14	1.69	2.03	1.62	5	S04	0.52	
yrbL	yrbL	STM3325	1.14E-03	0.00E+00	2.29E-03	1.14	1.51	1.92	1.52	TRUE	S01	0.90	
yeiU	yeiR	STM2213		0.00E+00	0.00E+00	0	1.87	2.54	1.47	>10	S01	0.63	
nmpC	nmpC	STM1572	1.05E-03	5.01E-03	5.05E-03	1.11	1.31	1.72	1.38	1	S02	0.89	
phoP	phoP	STM1231		0.00E+00	0.00E+00	0	1.73	2.25	1.33	>10	S01	0.88	
yjeH	yjeH	STM4328	0.00E+00	0.00E+00	3.16E-02	1	1.34	1.27	1.20	3	S01	0.56	
slyB	slyB	STM1445		3.51E-03	1.50E-03	0	1.42	2	1.14	>10	S02	0.85	
STM0271	STM0271	STM0271	6.77E-03	8.31E-03	3.16E-02	0.98	1.15	1.27	1.13	1	S09	0.57	
mig-14	mig-14	STM2782	0.00E+00		1.50E-03	1.36	0	2	1.12	>10	S05	0.97	
gst	gst	STM1451			1.50E-03	0	0	2.45	0.82	6	S10	0.52	
ompX	ompX	STM0833	5.47E-03	3.29E-03		1.03	1.4	0	0.81	TRUE	S12	0.94	
yhaO	yhaO	STM3239		2.30E-02	8.65E-02	0	1.04	1.18	0.74	1	S08	0.84	
pagP	pagP	STM0628		1.63E-02	1.59E-01	0	1.03	0.93	0.65	>10	S03	0.81	
STM2780	STM2780	STM2780	0.00E+00			1.82	0	0	0.61	6	S11	0.92	
ugtL	ugtL	STM1601	7.85E-02	3.77E-02		0.79	0.96	0	0.58	3	S06	0.73	
mgtC	mgtC	STM3764		7.98E-02	1.81E-01	0	0.85	0.9	0.58	TRUE	S08	0.87	
STM1939	STM1939	STM1939		7.09E-03		0	1.23	0	0.41	>10	S03	0.57	
ycfD	ycfD	STM1229		8.31E-03		0	1.15	0	0.38	8		--	
ybbS	ybbS	STM0514			6.53E-02	0	0	1.13	0.38	3	S01	0.57	
mdoB	mdoB	STM4541			7.38E-02	0	0	1.1	0.37	>10	S10	0.65	
pmrD	pmrD	STM2304		2.66E-02		0	1.03	0	0.34	>10	S11	0.88	
STM2714	STM2714	STM2714		3.77E-02		0	0.96	0	0.32	1	S07	0.50	
STM0081	STM0081	STM0081			1.59E-01	0	0	0.93	0.31	4		--	
STM2728	STM2728	STM2728			7.89E-02	0	0	0.93	0.31	>10	S10	0.65	
pipD	pipD	STM1094			1.81E-01	0	0	0.9	0.30	5	S06	0.83	
rna	rna	STM0617		7.98E-02		0	0.86	0	0.29	8		--	
pagK	pagK	STM1867		9.89E-02		0	0.82	0	0.27	5	S06	0.72	
ybjY	ybjY	STM0941		0.00E+00		0	1.88	0	0.63	1	S03	0.91	
ybiF	ybiF	STM0832	2.29E-03	3.29E-03	4.31E-03	1.06	1.4	1.81	1.42	8	S12	0.94	
STM1255	STM1255	STM1255	0.00E+00	0.00E+00	0.00E+00	1.41	1.76	2.14	1.77	1	S01	0.91	
aspS	aspS	STM1901	0.00E+00	0.00E+00	0.00E+00	1.55	1.74	2.31	1.87	4	S11	0.57	

*: Expression is computed by the number of probe sets with change fold >4 that overlap the gene location

Expression & No binding

Gene	Operon	Code	FDR ChIP 1	FDR ChIP 2	FDR ChIP 3	Peak ChIP 1	Peak ChIP 2	Peak ChIP 3	Average	*Expression	Submotif	Score	Notes
pagD	pagD	STM1244								>10	S05	0.87	
pagC	pagC	STM1246								>10	S05	0.87	
pdgL	pdgL	STM1599								>10	S10	0.79	
yobG	yobG	STM1840								>10	S08	0.66	
udg	udg	STM2080								>10	S03	0.78	
pgtE	pgtE	STM2395								4	S08	0.57	
phoN	phoN	STM4319								>10	S07	0.58	
hemL	hemL	STM0202								TRUE	S07	0.56	
hilA	hilA	STM2876								TRUE	S04	0.65	
yaiB	yaiB	STM0383								1	S03	0.80	
proP	proP	STM4290								5	S05	0.72	
STM2585	STM2585	STM2585								>10	S05	0.88	
STM2585A	STM2585A	STM2585A								5	S06	0.70	
STM2245	STM2245	STM2245								2	S11	0.82	
nagB	nagB	STM0684								TRUE	S03	0.68	
orgB	orgB/prgH	STM2870								>10	S06	0.82	
nagA	nagB	STM0683								TRUE			*within operon
STM2868	orgB/prgH	STM2868								>10			*within operon
orgA	orgB/prgH	STM2869								>10			*within operon
phoQ	phoP	STM1230								>10			*within operon
yoaE	STM1829	STM1828								>10			*within operon
prgH	prgH	STM2874								>10			*Indirect (orgB)
yfbE	yfbE	STM2297								>10			*Indirect (pbgP)
pmrF	yfbE	STM2298								>10			*Indirect (pbgP)
yfbG	yfbE	STM2299								>10			*Indirect (pbgP)
STM2300	yfbE	STM2300								>10			*Indirect (pbgP)
pqaB	yfbE	STM2301								>10			*Indirect (pbgP)
STM2302	yfbE	STM2302								>10			*Indirect (pbgP)
STM2303	yfbE	STM2303								5			*Indirect (pbgP)
basS	yjdB	STM4291								>10			*indirect (pmrC)
basR	yjdB	STM4292								>10			*indirect (pmrC)
yjdB	yjdB	STM4293								>10			*indirect (pmrC)
yeiR	yeiR	STM2212								>10			
STM1253	STM1253	STM1253								>10			
STM1269	STM1269	STM1269								>10			
STM1583	STM1583	STM1583								>10			
ybjD	ybjD	STM0939								8			

Expression & No binding

Gene	Operon	Code	FDR ChIP 1	FDR ChIP 2	FDR ChIP 3	Peak ChIP 1	Peak ChIP 2	Peak ChIP 3	Average	*Expression	Submotif	Score	Notes
STM0033	STM0033	STM0033								>10			
STM0212	STM0212	STM0212								>10			
STM0306	STM0306	STM0306								>10			
STM1839	STM1839	STM1839								>10			
STM1864	STM1863	STM1864								>10			
STM1940	STM1940	STM1940								>10			
ais	ais	STM2296								>10			
yfiA	yfiA	STM2665								>10			
STM2724	STM2724	STM2724								>10			
STM3595	STM3595	STM3595								>10			
ushB	ushB	STM4064								>10			
yjbA	yjbA	STM4226								>10			
STM2710	STM2710	STM2710								>10			
yicE	yicE	STM3747								8			
STM2701	STM2701	STM2701								6			
STM3126	STM3126	STM3126								6			
ompN	ompN	STM1473								4			
STM1863	STM1863	STM1863								4			
pqaA	pqaA	STM1544								3			
ybjG	ybjG	STM0865								2			
STM1548	STM1548	STM1548								2			
STM1854	STM1854	STM1854								2			
STM2734	STM2734	STM2734								>10			
STM2703	STM2703	STM2703								>10			
STM2704	STM2703	STM2704								3			
STM2738	STM2738	STM2738								>10			
STM2739	STM2738	STM2739								>10			
STM2737	STM2737	STM2737								2			
STM2736	STM2737	STM2736								2			
STM2706	STM2710	STM2706								>10			
STM2726	STM2728	STM2726								>10			
STM2727	STM2728	STM2727								5			
STM2697	STM2701	STM2697								4			
