# Supplementary material for: Defining the Plasticity of Transcription Factor Binding Sites by Deconstructing DNA Consensus Sequences: The PhoP-Binding Sites among Gamma/Enterobacteria
Source: PLoS Comput Biol. 2010 Jul 22;6(7):e1000862. doi: 10.1371/journal.pcbi.1000862 (PMC2908699; doi:10.1371/journal.pcbi.1000862)
Supplement: Table S7 — Genome-wide analysis of the S. typhimurium sequences using PhoP submotifs, gene expression and promoter occupancy of the PhoP protein. (0.04 MB PDF) [file pcbi.1000862.s012.pdf]

Table S7. Genome-wide analysis of the *S. typhimurium* sequences using PhoP submotifs, gene expression and promoter occupancy of the PhoP protein

			Gene			Operon		
	Expression	Chip	Submotif	Further valid.	No submotifs	Submotif	Further valid.	No submotifs
Coding/Intergenic	+	+	92%	8%	0%	92%	8%	0%
	+	-	23%	0%	77%	31%	0%	69%
	-	+	4%	15%	80%	4%	16%	80%
	-	-	1%	0%	99%	1%	0%	99%
Coding	-	+	5%	0%	95%	8%	0%	92%
