# Supplementary material for: Defining the Plasticity of Transcription Factor Binding Sites by Deconstructing DNA Consensus Sequences: The PhoP-Binding Sites among Gamma/Enterobacteria
Source: PLoS Comput Biol. 2010 Jul 22;6(7):e1000862. doi: 10.1371/journal.pcbi.1000862 (PMC2908699; doi:10.1371/journal.pcbi.1000862)
Supplement: Table S8 — Genome-wide analysis of Y. pestis using PhoP submotifs (0.35 MB PDF) [file pcbi.1000862.s013.pdf]

Table S8. Genome-wide analysis of *Yersinia* using PhoP submotifs

Operon	Gene	Fold	ChIP	Submotif	Binding Site
crcA(y2563)	crcA	5.2	●	S02/01	TCGTTATAGAACTGTTTAA
			○	S08	CAACTTTCACTTTGTTAAG
mgtC	mgtC	3.47	●	S03	TTGTTTAAGTATTGTTTAA
nqrA	nqrB	2.51	●	S11/09	CTGTTGACGTCCGGTTTAT
psiE	psiE	2.51	●	S12/09	GCGTTGATTAACCGTTTCA
			●	S04	CGGTTAGCCAGTTGTTTAC
slyB	slyB	2.65	●	S02	CCGTTTAATATTGGTTAAA
y0447	y0447	5.5	●	S02/01	CCGTTTATTTTTTGTTAAG
			○	S10	TCAAATACGAAGGGTATAT
y0838	y0838	5.36	●	S12/09	TTGTTGAGGATTGGTTGAT
y1306	y1306	2.51	●	S11	CCATTGCCAATGTCTTGAA
y1795	y1795	5.33	●	S03	CTGTTTACCCGCTGTTTAA
	phoP	5.47			
	phoQ	5.45			
	y1792	5.36			
y1877	y1877	5.48	●	S03/01	TTGTTTATAATTTGTTTAA
y1917(pbgp)	y1917	5.36	●	S02	GCGTTTAGTTTTCGTTAAC
	y1918	5.46			
	y1919	5.48			
	y1920	5.41			
	y1921	5.38			
	y1923	5.45			
	y1922	4.56			
y2124	y2124	5.4	●	S12	TTGTTGATGTTTAGTTTAA
y2147(ugd)	y2147	5.48	●	S03	TTGTTTATAAATGGTTTTA
y2608	y2608	5.2	●	S10/08	ATATTTACCTGCCGTTTAC
			●	S12	ACGTTGAAAAAGCTTTTAT
y2815	y2816	5.49	●	S11	TTATTGATATTTTGTTTAG
y3284	y3284	4.88	●	S12	TGGTTGTGTTAGTGTTTAA
			○	S04	CGGTTAAGCCACTGTTTTG
y3808	y3808	4.9	●	S10/08	TCATTTAGCTACTGTTAAG
			○	S12	CTGATTGGTTTTAATTGAG
			○	S08	GCAATCAGCTAGTCAATTT
y3948	y3948	5.5	●	S12	AATTTGAGGTTACGTTGAT
y4116	y4116	2.51	●	S12	AAGTTGATGCTTTGTTTCA
y4125	y4125	2.51	●	S12	CGGTTCACTCTTGTTTAAG
y4126	y4126	2.51	●	S10	ATATTTACTCTCTGTTTAT
			○	S04	TGGTTATTTTTTATTTTAG
			○	S10	TTTTTTAGAGACTGTGTTA
			○	S03	ATAATTCCGGAATATTTTT
fruR	fruR	2.52	●	S03	ATGTCTATAGCCTGTTTAT
ibpA	ibpA	2.52	●	S11	CCCTTGCTAATCTATTTAG
ibpB	ibpB	2.52	●	S12/09	CTGTTTATACAACGTTAAT
y0838	y0839	4.01			
	y0840	2.51			
ompC	ompC	2.52			
tcaA1	tcaA1	4.08			
y0181	y0181	5.5			
y0566	y0566	4.61			
y0666	y0666	2.69			
y0961	y0961	5.26			
y0962	y0962	5.5			
Y1046	Y1048	3.16			
y1730	y1730	3.59			
y2934	y2934	5.47			
y2942	y2942	2.9			
y2943	y2943	4.04			
y2946	y2945	5.45			
	y2946	5.49			
y2948	y2947	5.47			
	y2948	5.44			
cstA	cstA	2.79			
fabB	fabB	3.51			
y0239	y0239	2.51			
Y1055	Y1054	4.45			
	y1055	2.77			
y1579	y1580	2.51			
y1803	y1803	2.55			
y1878	y1878	3.03			
y2814	y2814	2.51			
y2858	y2858	3.88			
y2859	y2859	5.09			
y2868	y2868	2.52			
y3093	y3093	4.96			
y3555	y3553	5.25			
	y3554	3.4			
fadL	fadL	4.04			

○ Dnase I footprinted
