# Supplementary material for: Defining the Plasticity of Transcription Factor Binding Sites by Deconstructing DNA Consensus Sequences: The PhoP-Binding Sites among Gamma/Enterobacteria
Source: PLoS Comput Biol. 2010 Jul 22;6(7):e1000862. doi: 10.1371/journal.pcbi.1000862 (PMC2908699; doi:10.1371/journal.pcbi.1000862)
Supplement: Table S9 — CRP classifier using single motif and distances between CRP and RNAP BSs. (*) CC: Correlation Coeffient; SCC: Standardized Correlation Coefficient. (0.14 MB PDF) [file pcbi.1000862.s014.pdf]

Table S9. CRP classifier using single motif and distances between CRP and RNAP BSs

	Activators			Repressors			Activators & Repressors		
	SCC	SP	SN	SCC	SP	SN	SCC	SP	SN
**Single motif (SM)**	0.589	0.783	0.806	0.550	0.837	0.708	0.560	0.809	0.750
**Global distances & SM**	0.656	0.783	0.871	0.617	0.898	0.708	0.625	0.838	0.787
**CRP distances & SM**	0.710	0.803	0.903	0.664	0.906	0.750	0.644	0.842	0.801
