# Supplementary material for: Defining the Plasticity of Transcription Factor Binding Sites by Deconstructing DNA Consensus Sequences: The PhoP-Binding Sites among Gamma/Enterobacteria
Source: PLoS Comput Biol. 2010 Jul 22;6(7):e1000862. doi: 10.1371/journal.pcbi.1000862 (PMC2908699; doi:10.1371/journal.pcbi.1000862)
Supplement: Table S10 — CRP classifier using submotifs and distances between CRP and RNAP BSs. (*) CC: Correlation Coeffient; SCC: Standardized Correlation Coefficient. (0.14 MB PDF) [file pcbi.1000862.s015.pdf]

Table S10. CRP classifier using submotifs and distances between CRP and RNAP BSs

	Activators			Repressors			Activators & Repressors		
	SCC	SP	SN	SCC	SP	SN	SCC	SP	SN
**Single motif**	0.589	0.783	0.806	0.585	0.902	0.667	0.560	0.809	0.750
**Global distances**	0.745	0.874	0.871				0.747	0.894	0.853
**CRP distances**	0.748	0.843	0.903	0.845	0.929	0.917	0.753	0.913	0.838
