# Supplementary figures and images for: Defining the Plasticity of Transcription Factor Binding Sites by Deconstructing DNA Consensus Sequences: The PhoP-Binding Sites among Gamma/Enterobacteria
Source: PLoS Comput Biol. 2010 Jul 22;6(7):e1000862. doi: 10.1371/journal.pcbi.1000862 (PMC2908699; doi:10.1371/journal.pcbi.1000862)

A

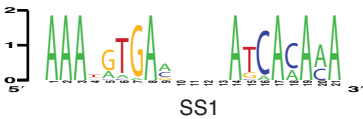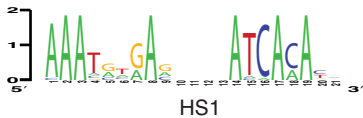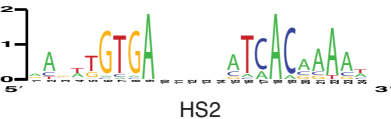

B

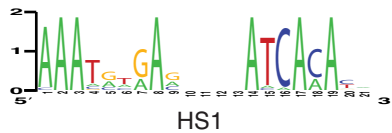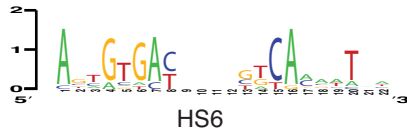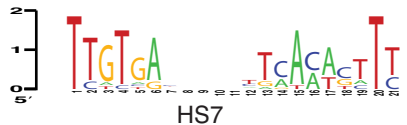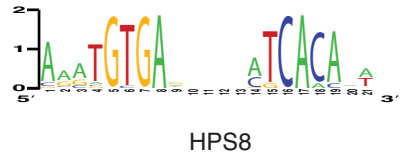

Supplement: Figure S1 — Intrinsic properties of the clustering algorithms recover distinct CRP Submotifs. A) The Subtractive Submotif 1 (SS1) groups 58 BSs that are assigned to two disjoint submotifs by the hierarchical algorithm: HS1, which has a total of 47 BSs; and HS2, which has a total of 32 BSs. Although there is a high degree of inclusion between SS1 and HS1 (p-value = 8.40E-04) and between SS1 and HS2 (p-value = 6.10E-03), the submotifs exhibit different patterns. SS1 encodes a general pattern that shows a balanced conservation between both tandems. In contrast the HS1 and HS2 exhibit more specific patterns with higher conservation of the second and first tandems respectively. C) Hierarchical possibilistic submotif 8 (HPS8) includes a mixture of 15 sequences from HS1 (p-value = 0.59), 3 sequences from HS6 (p-value = 0.59), and 3 sequences from HS7 (p-value = 0.53). Although the intersection of HPS8 cluster to the above clusters is not significant, its corresponding patterns shares with HS1 the second tandem, with HS6 the “GTGA” sequence; and with HS7 the “TGT-A” sequence. (0.07 MB PDF) [file pcbi.1000862.s016.pdf]

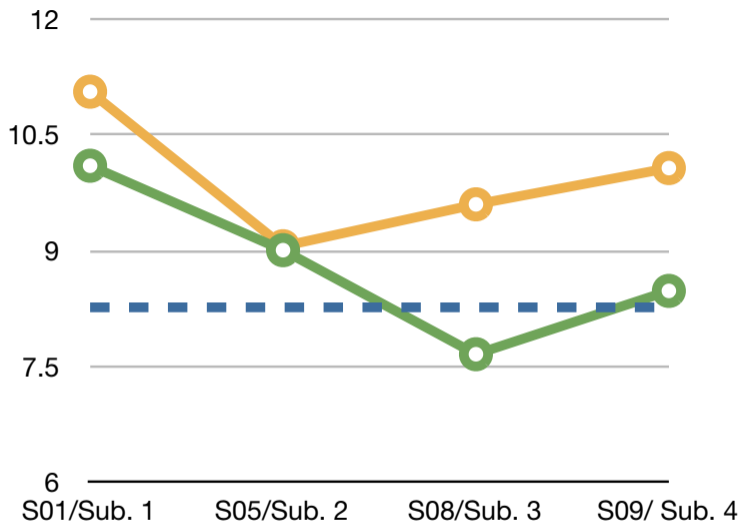

— Single Motif  
○ Hierarchical Possibilistic

○ Subtractive (Sub.)

Supplement: Figure S3 — Information content of PhoP submotifs. The four most general submotifs detected by the hierarchical possibilistic method (orange) exhibit a higher information content than those learned by the subtractive (green) and the single motif method (dashed blue). (0.06 MB PDF) [file pcbi.1000862.s018.pdf]

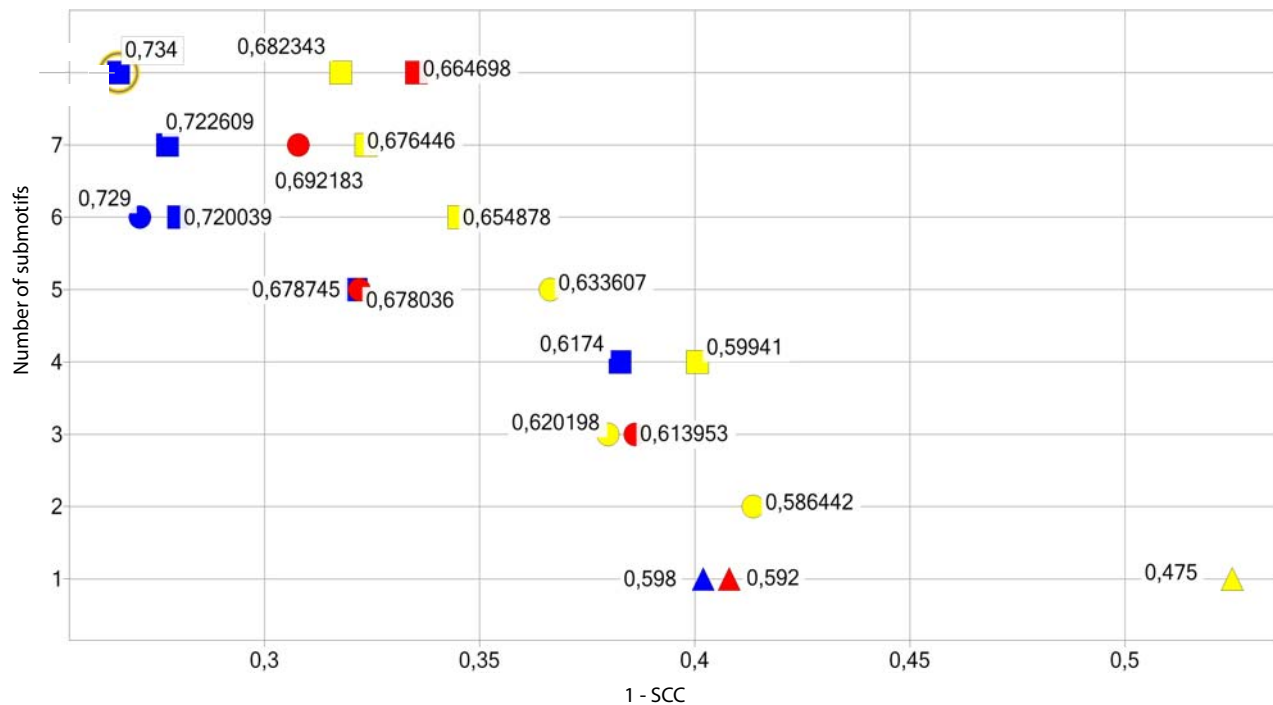

Color: ■ AlignAce ■ Consensus ■ MEME

Shape: ■ Hierarchical ● Subtractive ▲ Single Motif

Supplement: Figure S4 — Comparison among of different clustering and PWM methods uncovering CRP submotifs. Sets of submotifs generated by different clustering (shapes) and PWM (colors) methods are evaluated by their complexity (Y axis) and their performance, where the SCC metric was displayed as 1-SCC across the X axis. We identified all optimal solutions lying in the Pareto optimal frontier [9]. This frontier is the collection of multi-objective optima in the sense that its members are not worse than (i.e., dominated by) other solutions in any of the objectives being considered. (0.07 MB PDF) [file pcbi.1000862.s019.pdf]

A

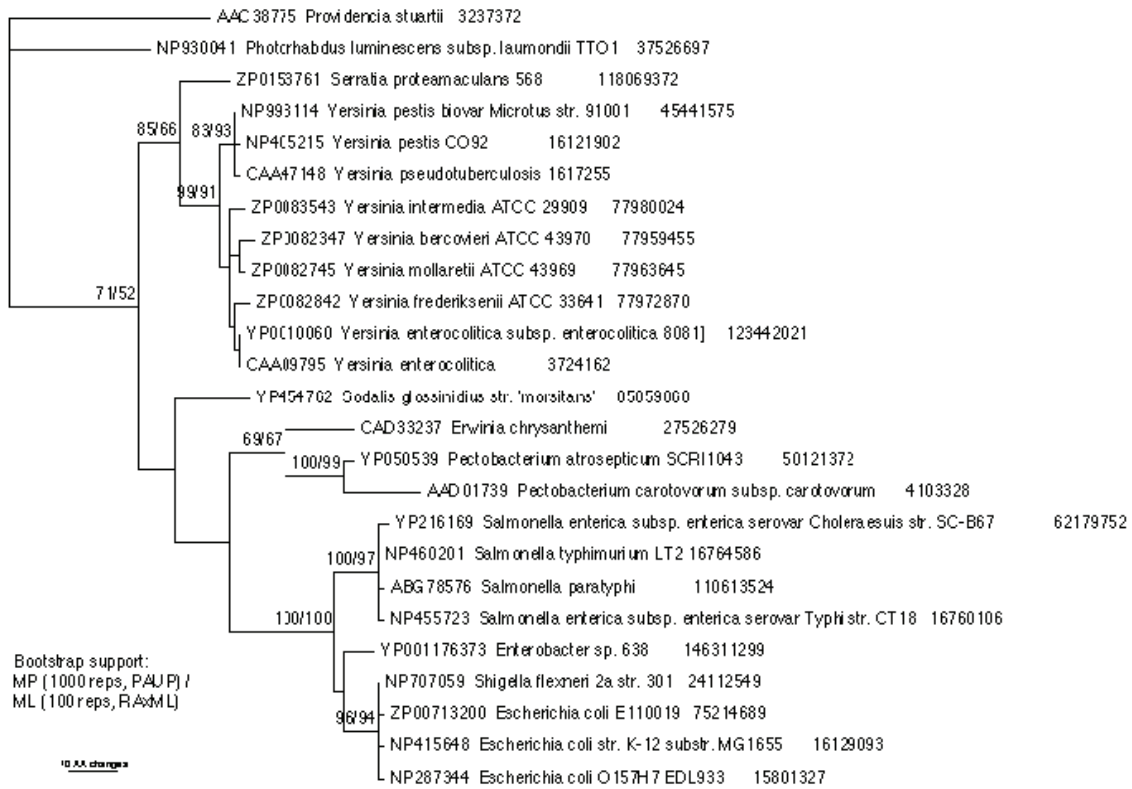

[illegible]

Supplement: Figure S6 — Evolution of the PhoP protein. A) Tree indicating the phylogenetic relationship among the PhoP protein for members of gama/enterobacterias. B) Alignment of the PhoP DNA-binding domain. (0.56 MB PDF) [file pcbi.1000862.s021.pdf]

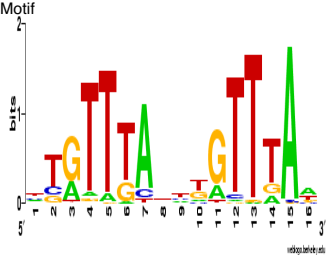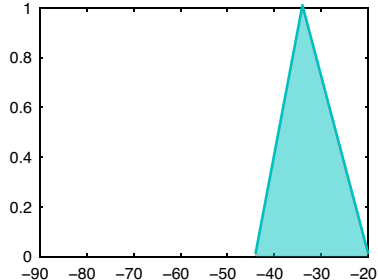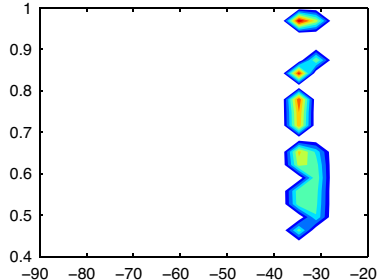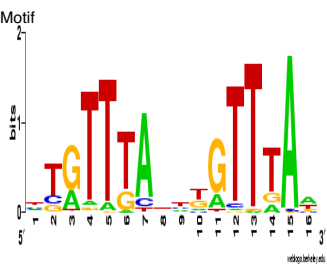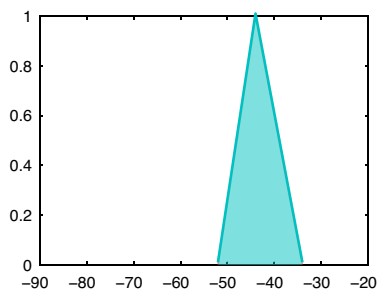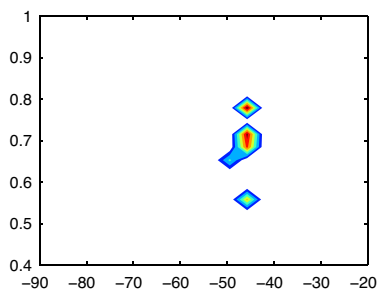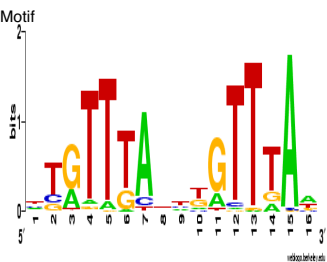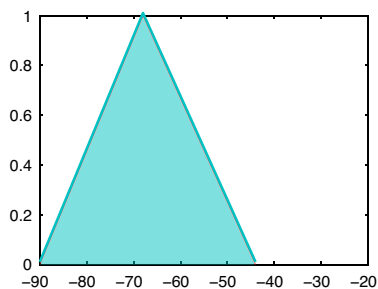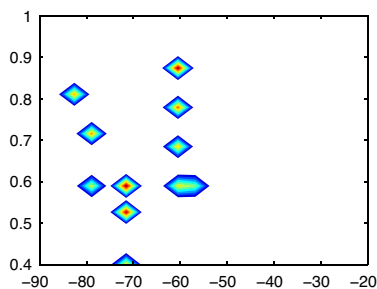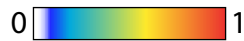

Supplement: Figure S7 — IF-THEN rules encompassing PhoP single motif and distances between PhoP BSs and RNAP BSs in activated promoters. Rows correspond to IF-THEN rules. The antecedent of the rules is composed of a single motif (left panel) and the distances between PhoP BSs and RNAP BSs (middle panel). The single motif is encoded as a fuzzy set based on their score distributions. The distances are approximated by their distributions (close, medium, and far distances from left to right panels), and also encoded by fuzzy sets. Both antecedents are combined a fuzzy AND operator (i.e., product). The consequent of a rule classifies PhoP BSs in the unit interval as a function of the antecedents (1: high, 0: low). Rules are activated concurrently, when they exceed each rule-specific threshold. The isobars show the degree of membership of the training set to the rules (red: high; blue: low). (0.06 MB PDF) [file pcbi.1000862.s022.pdf]

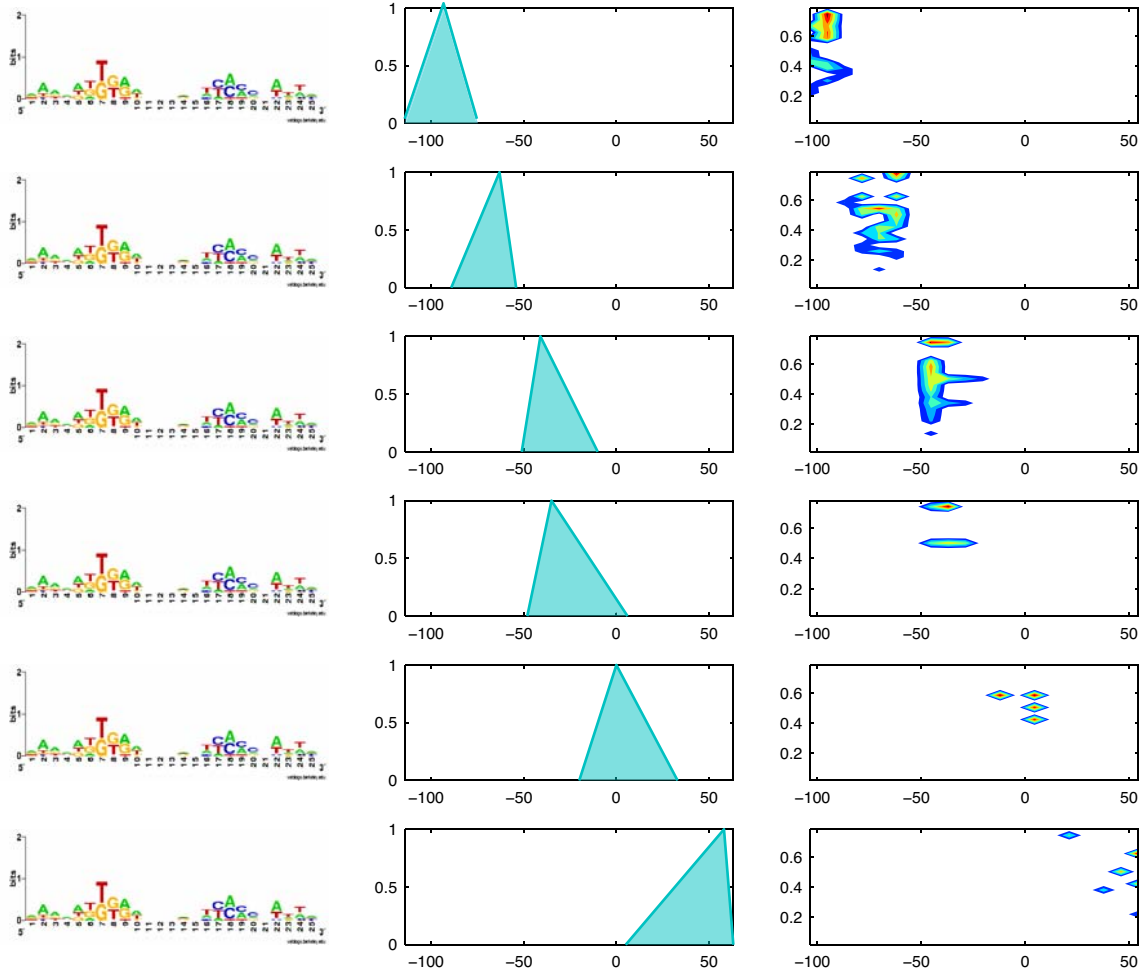

Supplement: Figure S8 — IF-THEN rules encompassing CRP submotifs and distances between CRP BSs and RNAP BSs. Rows correspond to IF-THEN rules. The antecedent of the rules is composed of submotifs (left panel) and the distances between PhoP BSs and RNAP BSs (middle panel). The submotifs are encoded as fuzzy sets based on their score distributions. The distances are approximated by their distributions (close, medium, and far distances from left to right panels), and also encoded by fuzzy sets. Both antecedents are combined a fuzzy AND operator (i.e., product). The consequent of a rule classifies PhoP BSs in the unit interval as a function of the antecedents (1: high, 0: low). Rules are activated concurrently, when they exceed each rule-specific threshold. The isobars show the degree of membership of the training set to the rules (red: high; blue: low). (0.07 MB PDF) [file pcbi.1000862.s023.pdf]

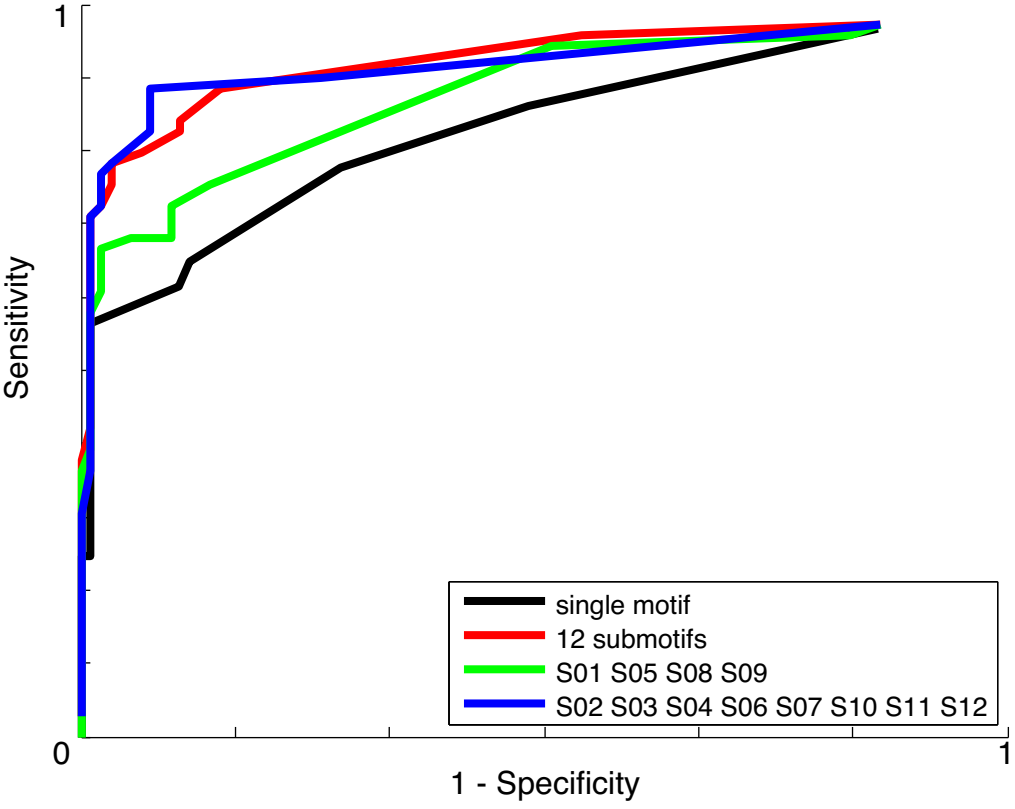

Supplement: Figure S9 — ROC curves comparing the performance of classifiers based on submotifs and single motif. ROC curves corresponding to the single motif classifier (black line), and to multi-classifiers using all submotifs (red line), the most specific set of submotifs (blue line), and the most general set of submotifs (green line). The performance of the single motif is outperformed by all multi-classifiers using submotifs. (0.03 MB PDF) [file pcbi.1000862.s024.pdf]
