# Supplementary material for: Defining the Plasticity of Transcription Factor Binding Sites by Deconstructing DNA Consensus Sequences: The PhoP-Binding Sites among Gamma/Enterobacteria
Source: PLoS Comput Biol. 2010 Jul 22;6(7):e1000862. doi: 10.1371/journal.pcbi.1000862 (PMC2908699; doi:10.1371/journal.pcbi.1000862)
Supplement: Figure S2 — PhoP submotifs learned by the subtractive clustering method. Three PhoP submotifs generated by the subtractive clustering. Their incorporation into a multi-classifier improved SCC by 33% (i.e., 0.73 vs. 0.547) and CC by 25% (i.e., 0.822 vs. 0.653) with respect to the single motifs. Visually, the patterns revealed slight differences among them, resulting in a decreasing level of nucleotide conservation (i.e., 13.84; 9.72 and 6.76 information content, respectively). Only one significant (p-value = 0.002) coincidence exist among the submotifs generated by the subtractive and the hierarchical possibilistic clustering methods (i.e., 6 of the 11 BSs forming the SS1 submotif coincide with 6 of the 13 BSs forming the S03 submotif). (0.06 MB PDF) [file pcbi.1000862.s017.pdf]

Group	Logo	Gene	Sequence	Organism
Subtractiive 1	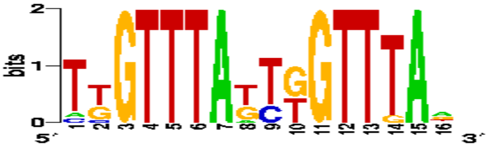	mgtA:	TGGTTTAT---TGGTTTAG	*E. Coli*
		mgtA:	TGGTTTAT---TGGTTTAA	*Salmonella*
		ompT:	CTGTTTAT---TTGTTTAG	*E. Coli*
		phoP:	TGGTTTAT---CTGTTTAT	*Salmonella*
		slyB:	TTGTTTAT---TGGTTGAT	*E. Coli*
		treR:	TGGTTTAT---TGGTTTAG	*E. Coli*
		udg:	ATGTTTAA---CGGTTTAA	*Salmonella*
		ybcU:	TTGTTTAG---TTGTTTAA	*E. Coli*
		ybjX:	TTGTTTAG---CGGTTTAC	*Salmonella*
		yrbL:	TTGTTTAG---TTGTTTAA	*E. Coli*
		yrbL:	TCGTTTAG---TTGTTTAA	*Salmonella*
Subtractive 2	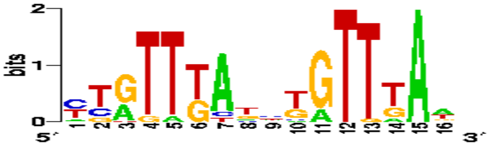	b1826:	ATAGTTAG---CTGTTTAA	*E. Coli*
		hdeA:	CTGTATAT---ATGTTGAT	*E. Coli*
		hdeD:	CTGTATAT---ATGTTGAT	*E. Coli*
		irap2:	TTGTTTTT---TGGTTTAT	*Salmonella*
		mgtC:	TTAGTGAC---CTGTTTAA	*Salmonella*
		mig-14:	ATGTTTAG---GTATTTAA	*Salmonella*
		nagA:	CTGTTTAT---CGGTGTAG	*E. Coli*
		nmpC:	TTATTTTT---CGGTTTAA	*E. Coli*
		nmpC:	TCGTTTAA---TGGTTGAG	*Salmonella*
		ompT:	ATATTGCT---CTGTTTAT	*E. Coli*
		ompX:	TGGTTTAT---TGGTTTAG	*E. Coli*
		ompX:	CGGTTGAG---TCGTTGAA	*Salmonella*
		pagC:	TTATTTAC---GTGTTTAA	*Salmonella*
		pagK:	CCATTTAT---ATATTTAA	*Salmonella*
		pagP:	CTGTTTAT---TTGTTAAG	*Salmonella*
		phoP:	TGGTTTAT---ATGTTTAC	*E. Coli*
		pmrD:	CCGTTGAT---GAGTTTAT	*E. Coli*
		pmrD:	CTATTGCC---TTGTTTAT	*Salmonella*
		rstA:	TTGTTTAG---CGATTGAT	*E. Coli*
		rstA:	TCGTTTAG---AGATTTAT	*Salmonella*
		slyB:	TCGTTTAA---TGGTTAAT	*Salmonella*
		trs5:	GTTTTTAA---CTGTTTAA	*Salmonella*
		ugtL:	CGGTTGAG---CTATTTAC	*Salmonella*
		virK:	CCATTGAT---CTGTTTAA	*Salmonella*
		ybjX:	TTGTTGAT---TCGTTGAA	*E. Coli*
		ybjX:	CTATTGAT---TGGTTAAG	*E. Coli*
Subtractive 3	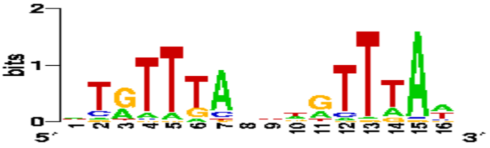	hemL:	ATGTTTGA---GTATTTAA	*E. Coli*
		mgtC:	ATGTTTAA---CGCTTTAT	*Salmonella*
		mgtC:	CTGTTTAA---TGTTTGAT	*Salmonella*
		mgtC:	AATAATAC---TAGTTTAA	*Salmonella*
		pagC:	GTGTTTAG---GAATTTAC	*Salmonella*
		pagD:	GTGTTTAG---GAATTTAC	*Salmonella*
		pdgL:	ATTTTAAC---CTGTTTAA	*Salmonella*
		pgtE:	ATGATTAT---TTGCTTAT	*Salmonella*
		pipD:	TTATTGAG---GTATTGAT	*Salmonella*
		proP:	TCGTTTAG---TCATTGAT	*E. Coli*
		virK:	AAGTTGAT---AAATTTAA	*Salmonella*
		ybjG:	TTCTTTAA---TTATTTAA	*E. Coli*
		ybjX:	ATATTTCG---AAGTTAAT	*E. Coli*
		ybjX:	GTATTGAC---TGGTTAAT	*Salmonella*
		yeaF:	TTGTTTAA---ATGATTAA	*E. Coli*
		yhiW:	GCGTATAG---ATGTTTAT	*E. Coli*
		yiaG:	TTGTTGTT---TTGTTAAA	*E. Coli*
