# Supplementary material for: Defining the Plasticity of Transcription Factor Binding Sites by Deconstructing DNA Consensus Sequences: The PhoP-Binding Sites among Gamma/Enterobacteria
Source: PLoS Comput Biol. 2010 Jul 22;6(7):e1000862. doi: 10.1371/journal.pcbi.1000862 (PMC2908699; doi:10.1371/journal.pcbi.1000862)
Supplement: Figure S5 — Optimal configurations of PhoP submotifs encoded into PWM using A) MEME and B) AlignACE. GA optimization was applied on the number and thresholds of submotifs (CF). The fitness function was calculated by either SCC or CC measurements (OO). Different selection pressures (SN) where used as initial constrains (See parameter in Materials and Methods). TP/TN and FP/FN stand for true/negative and positive/negative predicted values, respectively. #Sub indicates the number of submotifs effectively employed, columns S1 to S12 represent the submotifs organized as families. Dots at the columns (black: general submotif; white: specific submotif) indicate that the corresponding submotif was selected by the optimization process for that configuration (rows). Min Th. corresponds to the minimum learned threshold. SM shows the results obtained by the single motif. (0.06 MB PDF) [file pcbi.1000862.s020.pdf]

A

MEME

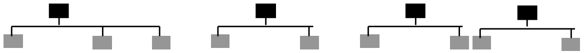

CF	OO	SN	TP	TN	FP	FN	CC	SCC	#Sub	S2	S1	S3	S4	S6	S5	S7	S10	S8	S11	S9	S12	Min Th.
	SM		38	770	2	31	0.707	0.613	1													0.55
1	CC	0	64	768	4	5	0.92851	0.92445	12	●	●	●	●	●	●	●	●	●	●	●	●	0.6831
2		0.1	60	770	2	9	0.91049	0.87415	9	●	●	●	●	●		●	●		●	●		0.5755
3		0.2	57	770	2	12	0.88484	0.83585	6		●	●	●	●			●		●	●		0.6558
4		0.3	58	768	4	11	0.87733	0.84552	4			●			●		●			●		0.5754
5	SCC	0	66	756	16	3	0.86574	0.93604	12	●	●	●	●	●	●	●	●	●		●	●	0.0593
6		0.1	62	758	14	7	0.84272	0.88349	9	●	●	●	●	●		●	●		●	●		0.6503
7		0.2	59	764	8	10	0.85612	0.85246	7	●		●	●		●	●		●	●	●		0.6770
8		0.3	58	768	4	11	0.87733	0.84552	4			●			●		●			●		0.5754

B

AlignACE

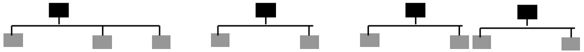

CF	OO	SN	TP	TN	FP	FN	CC	SCC	#Sub	S2	S1	S3	S4	S6	S5	S7	S10	S8	S11	S9	S12	Min Th.
	SM		48	759	13	21	0.718	0.709	1													0.65
1	CC	0	59	766	6	10	0.87073	0.85538	12	●	●	●	●	●	●	●	●	●	●	●	●	0.6875
2		0.1	57	766	6	12	0.85309	0.82985	10		●	●	●	●	●	●	●	●	●		●	0.6211
3		0.2	57	765	7	12	0.8456	0.82835	6		●	●	●	●			●		●	●		0.6710
4		0.3	57	761	11	12	0.81726	0.82239	4			●			●		●			●		0.6532
5	SCC	0	62	758	14	7	0.84272	0.88349	12	●	●	●	●	●	●	●	●	●		●	●	0.6211
6		0.1	60	762	10	9	0.85102	0.86259	10	●	●	●	●	●	●	●	●	●	●	●		0.6656
7		0.2	59	765	7	10	0.86334	0.85392	7		●	●		●	●		●	●			●	0.6686
8		0.3	60	750	22	9	0.7781	0.84547	4			●			●		●			●		0.5327
